# Contributions to the Taxonomy of *Arlesminthurus* Bretfeld and *Calvatomina* Yosii (Collembola, Symphypleona, Appendiciphora), with the Description of New Species from Northeastern Brazil †

**DOI:** 10.3390/insects12050433

**Published:** 2021-05-11

**Authors:** Nikolas G. Cipola, Gleyce Da S. Medeiros, Viviane A. M. De Oliveira, Luís G. De M. Barbosa, Thainá A. Lycarião, Bruno C. Bellini

**Affiliations:** 1 Laboratório de Sistemática e Ecologia de Invertebrados do Solo, Instituto Nacional de Pesquisas da Amazônia—INPA, CPEN, Avenida André Araújo, 2936, Aleixo, Manaus 69067-375, Brazil; nikolasgc@gmail.com; 2 Laboratório de Collembola, Departamento de Botânica e Zoologia, Centro de Biociências, Universidade Federal do Rio Grande do Norte—UFRN, Lagoa Nova, Campus Universitário, Natal 59072-970, Brazil; vivianeaurora0101@gmail.com (V.A.M.D.O.); luisgustavo.dsb@gmail.com (L.G.D.M.B.); 3 Universidade Estadual da Paraíba—UEPB, Campus IV, Sítio Cajueiro—Zona Rural, Catolé do Rocha 58884-000, Brazil; thain4h@gmail.com

**Keywords:** bothriotrichum-like sens, Bourletiellidae, Bourletiellinae, chaetotaxy, Dicyrtomidae, Dicyrtominae, identification keys, Neotropical springtails

## Abstract

**Simple Summary:**

Springtails (Hexapoda, Collembola) are tiny insect-like animals found mostly in soil habitats. Symphypleona is one of the four orders of springtails, gathering species with rounded bodies and antennae longer than the head. Here, we describe in detail two new species of Neotropical Collembola, both from northeastern Brazil: *Arlesminthurus caatinguensis* sp. nov. and *Calvatomina gladiata* sp. nov. We also provide detailed notes on their morphology, especially on their chaetotaxy (the shape and arrangement of the chaetae over the head, body and appendages), to give support to future studies on comparative anatomy, taxonomy and evolution of the Symphypleona.

**Abstract:**

*Arlesminthurus* Bretfeld is a small genus of Neotropical Bourletiellidae, with only four described species so far. *Calvatomina* Yosii is a widespread taxon of Dicyrtomidae, with most species known from the tropics. Here, we describe two new species from northeastern Brazil: *Arlesminthurus caatinguensis* sp. nov. and *Calvatomina gladiata* sp. nov. We also provide a detailed chaetotaxic study for *Arlesminthurus* for the first time, with updated diagnoses and identification keys for the Neotropical species of both genera and notes on their morphology. *Arlesminthurus caatinguensis* sp. nov. resembles *A. aueti* Arlé in body color pattern, male head and dental chaetotaxy. The discovery of one bothriotrichum-like sens on the large abdomen of the new species needs to be investigated as a possible generic diagnostic feature, but we suggest that this structure is homologous to the S-sens seen in at least four Bourletiellinae genera, and they are likely related to each other. *Calvatomina gladiata* sp. nov. belongs to the *rufescens*-group and resembles *C. rufescens* Reuter and *C. guyanensis* Nayrolles and Betsch in some aspects of the head, dental and abdominal chaetotaxy. These descriptions represent the first record of *Arlesminthurus* from Caatinga and the first nominal species of *Calvatomina* from Brazil.

## 1. Introduction

Symphypleona Börner (sensu Bretfeld [1]) is an order of springtails with global distribution and holding almost 1300 species in 122 genera and 10 families [2]. Of these, about 1100 species in 109 genera and eight families belong to the Appendiciphora [3], a group which is recognized by the presence of a pair of subanal appendages on the female’s small abdomen [2,4].

In Brazil, 71 nominal species of Symphypleona have been recorded so far, of which 46 species in 18 genera and seven families represent the Appendiciphora [5]. Of these families in the country, Bourletiellidae is represented by only eight species in five genera and Dicyrtomidae by three species in one single genus [5]. Very little is known about these families, considering that they are among the most diverse and widespread Symphypleona, just after the Sminthuridae [2], and the fact that Brazil shelters several species-rich habitats.

An example of this lack of knowledge is seen in northeastern Brazil, where only 16 species of Symphypleona have been registered until now, two of Bourletiellidae and only one of Dicyrtomidae [5,6,7], but none of the genera *Arlesminthurus* Bretfeld, 1999 [4] (Bourletiellidae) or *Calvatomina* Yosii, 1966 [8] (Dicyrtomidae), although the latter has been recorded with non-nominal specimens in different regions from Brazil [7,9,10].

*Arlesminthurus* is a bourletiellid genus that is exclusively Neotropical, currently with four described species, three from Brazil and one from Nicaragua. Such species are usually associated with aquatic environments [5,11,12,13,14]. On the other hand, *Calvatomina* is the largest genus of Dicyrtominae, and it is widely distributed and holds 34 valid species, most of them from tropical areas, but with only five taxa recorded from the Neotropical region [2,15,16,17,18]. Although the genus may be widespread in Brazil, as already stated, there has been no nominal species of it recorded from the country until now. All known species of Brazilian dicyrtomids belong to *Ptenothrix* Börner, 1906 [2,5,19].

Herein, two new species, *Arlesminthurus caatinguensis* sp. nov. and *Calvatomina gladiata* sp. nov., are described from northeastern Brazil and illustrated in detail. For the new species of *Arlesminthurus* we provide a detailed map of the chaetotaxic homologies, the first one for the entire genus (for *Calvatomina* a similar chaetotaxic study was presented in Nayrolles and Betsch [18]). We also provide updated diagnoses to both genera, a key to all species of *Arlesminthurus* and another to the Neotropical taxa of *Calvatomina*, as well as comparative tables plus notes on the morphology of each genus.

## 2. Materials and Methods

Specimens preserved in ethanol (92% or 70%) were cleared in Nesbitt’s solution and then mounted on glass slides in Hoyer’s medium following the procedures described in Cipola et al. [14]. Maps of species localities were made after Shorthouse [20].

The type material was deposited at the Invertebrate Collection of the National Institute of Amazonian Research (INPA), Manaus, and at the Collembola Collection of Biosciences Center of Federal University of Rio Grande do Norte (CC/UFRN), Natal, Brazil.

The terminology used in descriptions follows mainly: antennal chaetotaxy after Nayrolles [21,22,23], labral chaetotaxy after Cipola et al. [24]; labial papillae and maxillary palp after Fjellberg [25], tibiotarsal chaetotaxy after Nayrolles [26], unguiculus lamellae after Hüther [27], tenaculum after Nayrolles [28] and furcula chaetotaxy after Nayrolles [29]. The chaetotaxy of the head and large abdomen was named after Betsch and Waller [30], and of the small abdomen after Betsch [31]. We also used, in the *Calvatomina* description, Nayrolles and Betsch [18] as an alternative depiction of the homologies in the small abdomen, to better compare the new species with previous described taxa. Chaetae labels, unguiculus lamellae and tenaculum structures are marked in bold in the text.

Abbreviations used in descriptions are: Ant―antennal segment(s), Abd―abdominal segment(s), Th―thoracic segment(s). Other abbreviations used only in the *Arlesminthurus caatinguensis* sp. nov. description are: for mouthparts: bc―basal chaeta(e), l.p.―lateral process of labial papilla E, lpc―labial proximal chaeta(e), t.a.―terminal appendix, sl.p.―sublobal plate; for unguiculus: ai―antero-internal lamella, ae―antero-external lamella, pi―postero-internal lamella, pe―postero-external lamella; for tenaculum: da―apical teeth, db―basal teeth, dm―median teeth, la―lobe anterior, tb―tubercule basal.

## 3. Results

### 3.1. *Taxonomic Summary of* Arlesminthurus *and Genus Diagnosis*

Order Symphypleona Börner, 1901 [32] sensu Bretfeld, 1986 [3]

Suborder Appendiciphora Bretfeld, 1986 [3]

Superfamily Sminthuroidea Bretfeld, 1994 [1]

Family Bourletiellidae Börner, 1913 [33] sensu Bretfeld, 1994 [1]

Subfamily Bourletiellinae Börner, 1913 [33] sensu Betsch, 1974a [34]

Genus *Arlesminthurus* Bretfeld, 1999 [4]

Diagnosis: Body predominantly with smooth and acuminate chaetae, males with some spines and spine-like chaetae on head interocular region and large abdomen dorsally, females only with smooth chaetae of different sizes. Males’ antennae longer than the females, in relation to their own body (Figure 4). Male interantennal region with two pairs of modified chaetae. Small abdomen with **E** bothriotrichum. Females’ subanal appendage simple and spine-like, smooth and acuminate. Tibiotarsi I–III with three, three and two capitate tenent hairs, respectively. Dens dorsal side weakly crenulate, inner and outer sides commonly with some elongated chaetae, mucro with both sides lamelled (Figure 17C) [4,11,12,13].

### 3.2. Arlesminthurus caatinguensis *sp. nov. Cipola, Lycarião and Medeiros*

Figure 1, Figure 2, Figure 3, Figure 4, Figure 5, Figure 6, Figure 7, Figure 8, Figure 9, Figure 10, Figure 11, Figure 12, Figure 13, Figure 14, Figure 15, Figure 16 and Figure 17.

**Figure 1:**
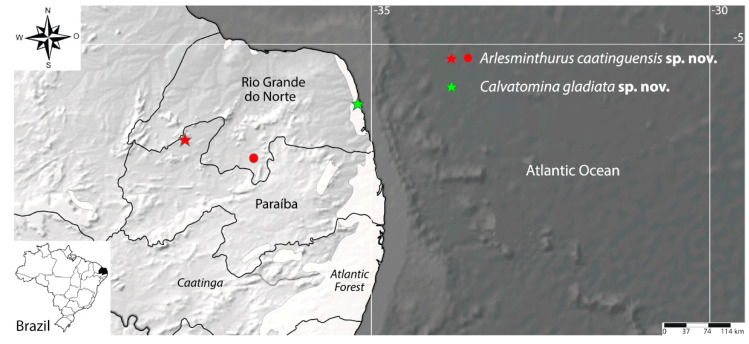
Map of the localities where *Arlesminthurus caatinguensis* sp. nov. and *Calvatomina gladiata* sp. nov. were found in northeastern Brazil; stars represent the type localities and the circle an additional locality for the first species.

**Figure 2:**
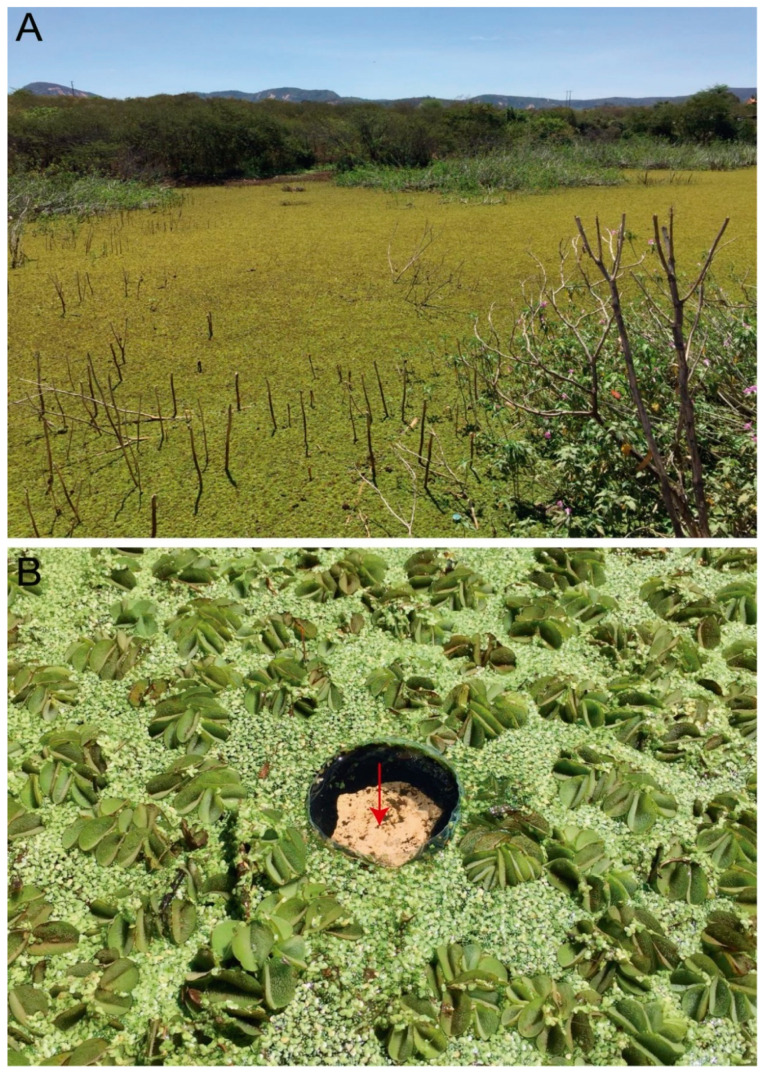
Habitat of the type locality of Arlesminthurus caatinguensis sp. nov.: (**A**) shallow wa-ters covered by macrophytes in Catolé do Rocha municipality, Paraíba State, Brazil; (**B**) pit-fall-traps on the water surface among Salvinia auriculata, Lemna minor and Wolffia sp.; red arrow indicates the stone inside the trap.

Type Material: Holotype male in slide (INPA-CLL 000117): Brazil, Paraíba state, Catolé do Rocha municipality, lagoon at the city west entrance, 06°20′53.9″ S, 37°45′37.8″ W (Figure 1), 287 m., 23–24 August 2020, pitfall-trap on the water surface among floating macrophytes such as *Salvinia auriculata, Lemna minor* and *Wolffia* sp. (Figure 2), TA Lycarião and NG Cipola coll. 22 paratypes: two males, two females, two subadult females and two juveniles on slides, plus 11 specimens in alcohol (INPA-CLL 000117); one male, one female and one juvenile in slides (CC/UFRN).

Other examined material: two males, four females and one juvenile on slides (CC/UFRN): Brazil, Rio Grande do Norte State, Jardim do Seridó municipality, “Zangarelhas” farm, 06°36′21.43″ S, 36°44′43.32″ W (Figure 1), 236 m., in the dry period, 11 March 2017, pitfall-trap, JS França and OJR Siqueira colls.

Diagnosis: Both sexes with the same color pattern, with head with a longitudinal strip, large abdomen anteriorly with two lateral spots and one longitudinal strip depigmented; Ant IV with eight subsegments, Ant III with two oval organ-like microsensilla on males and only one ventral in females, **Aai** chaeta absent; male’s head with 0–4 spines and 5–9 spine-like chaetae on the interocular region, frontal region with **γ** inferior chaeta short and robust and **γ** superior chaeta apically hooked, **f** line with the unpaired chaeta, if present, subequal to the others; male’s thoracic region with 1–4 spines and 2–3 spine-like chaetae, abdominal region with 13–15 spines and 13–16 spine-like chaetae; large abdomen inferiorly with one bothriotrichum-like sens; female’s small abdomen with one robust spine-like chaeta on the superior lobe; unguis inner tooth present; dens with eight chaetae on each side, with the exception of the ventral formula with 3,3,1,1…1 chaetae, outer side with eight chaetae somehow elongated, inner side with seven elongated chaetae; mucro wide and with two invaginations on both sides (inner and outer).

**Figure 3:**
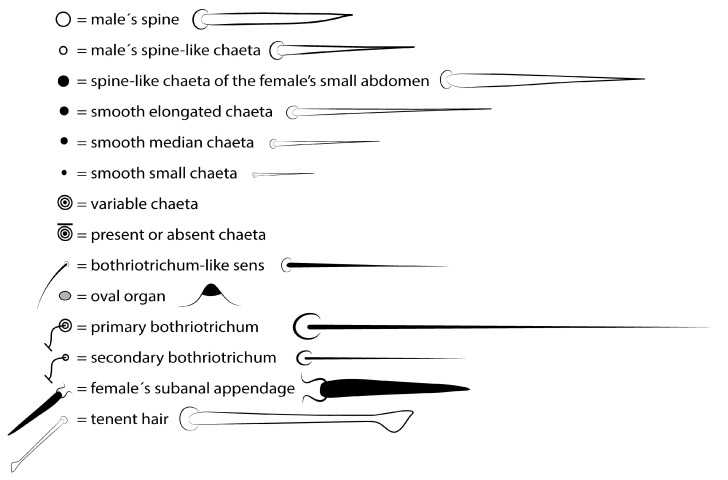
Chaetae symbols used in the chaetotaxy description of *Arlesminthurus caatinguensis* sp. nov.

**Figure 4:**
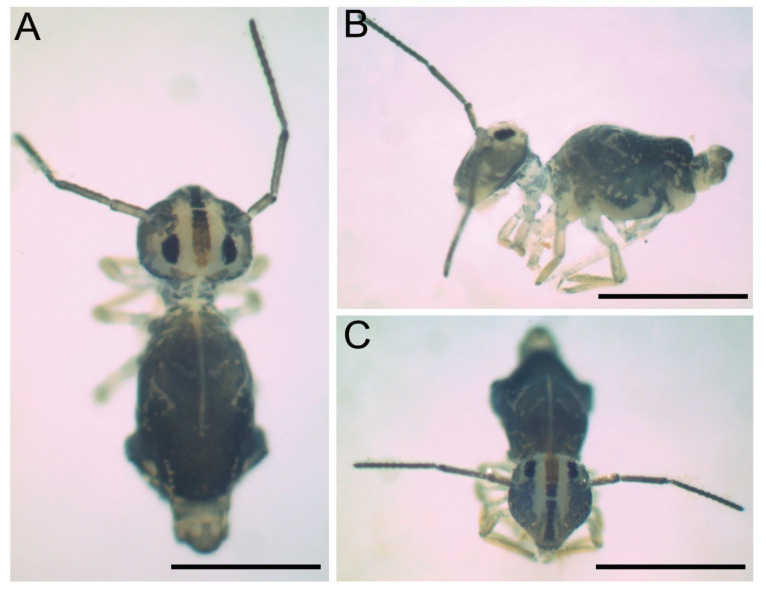
Habitus of *Arlesminthurus caatinguensis* sp. nov. fixed in ethanol, female: (**A**) dorsal view; (**B**) lateral view; (**C**) frontal view. Scale bars: 0.5 mm.

Description: Total length (head + large abdomen) of holotype 0.96 mm, in males 0.88–0.96 mm (*n* = 3), and females 1.12–1.13 (*n* = 3). Body with different types of chaetae as presented in Figure 3. Both sexes with the same color pattern. Frontal head with one longitudinal reddish strip from the anterior to the postocellar region, with a depigmented interruption in the subantennal region; body with dark bluish to greenish pigments on antennae, head (except clypeal part to postocellar and ventro-distal region) and on large and small abdomens (except ventrally). Large abdomen with depigmented patches and strips in specific patterns, such as a longitudinal line from the head to one third of the large abdomen, a circle followed by two transverse bands, and distally 5–6 small circles; laterally with about 10 depigmented circles (seven median and three distally). Legs with traces of pigments, mainly in the joints between the segments; eyepatches black (Figure 4).

**Figure 5:**
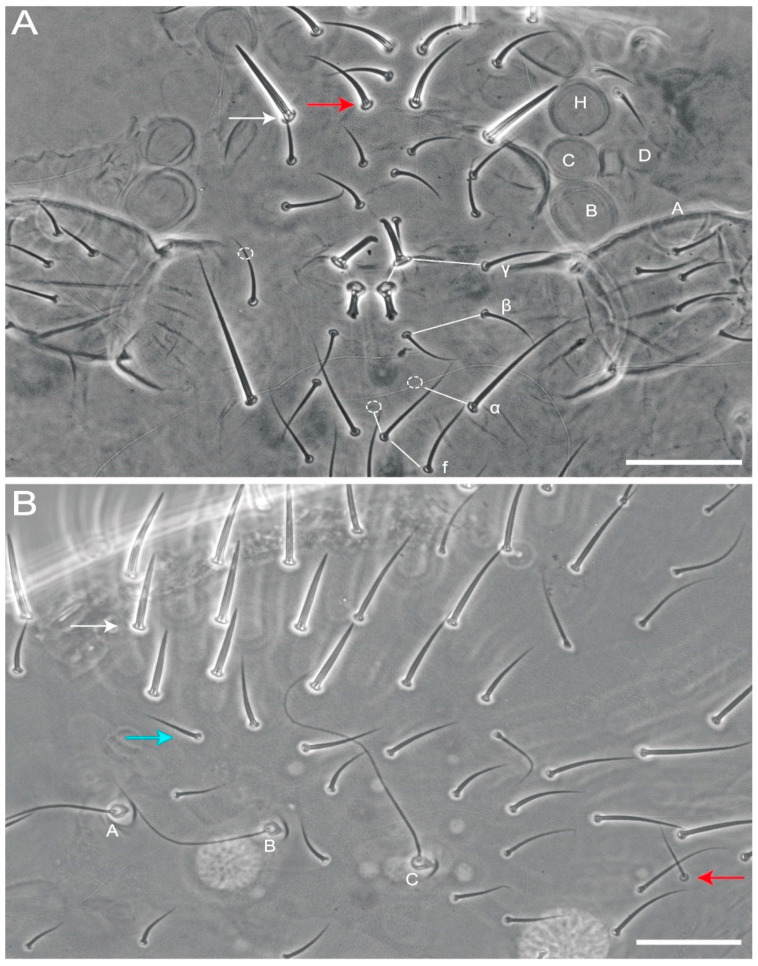
*Arlesminthurus caatinguensis* sp. nov., male structures. (**A**) Part of the head chaetotaxy (frontal, interocular and occipital regions); the white arrow shows a spine and the red arrow a spine-like chaeta; A–D and H represent the eyes; dashed circles indicate chaetae present or absent; white lines are clypeal (f) and frontal lines (α, β and **γ**). (**B**) Part of the large abdomen (lateral view); A–C represent the bothriotricha; the white arrow shows a spine; the blue arrow shows a spine-like chaeta; and the red arrow indicates an extra bothriotrichum-like sens on the inferior side. Scale bars: 0.04 mm.

Head (Figure 5A, Figure 7, Figure 8 and Figure 9): Antennae variable in size, slightly smaller or subequal to the large abdomen length in males (Figure 4 and Figure 7A), ratio antennae: large abdomen = 1:0.97 in holotype, in males as 1:0.84–0.97 (*n* = 3), and slightly longer in females as 1:1.17–1.19 (*n* = 3). Antennal segments ratio as I:II:III:IV = 1:2:3.6:5.17 in holotype, in males as 1:2–2.4:3.6–4.4:5.17–6.8, and females as 1:1.85–2.4:2.77–3.8:4.62–6.2. Ant IV with eight subsegments (A, M, B) (Figure 7A); A subsegment with a unilobed apical bulb (AI), one dorso-lateral microsensillum (AII), and 13 dorsal and 10 ventral chaetae, AI–III areas with eight, eight and nine chaetae, respectively (Figure 7A–C); M subsegment subdivided into six (M1–6), each with a single whorl of generally 10 chaetae, two latero-distal chaetae smaller (**Heae** ventrally, **Hppe** dorsally), both present or absent on M3 and M4, and **Heae** present or absent on M6; B subsegment with 33–40 chaetae, 7 BA, 21–28 BM (3–4 whorls with seven chaetae each) and five BB (**Gpi, Gi, Gai** absent) (Figure 7A,D,E). Ant III with 37 chaetae of different sizes, 23 dorsal and 14 ventral; distal whorl ventrally with two conical organs inside a single oval invagination, two oval organ-like microsensilla (one dorsal and one ventral, only one ventral in females), and six chaetae (**Ai, Api, Ap, Ape, Ae, Aa**), **Ai** slightly smaller, **Ap** smaller, and **Api** and **Ape** reduced; **Aai** absent, but possibly homologous to one of the ventral oval organ-like microsensilla (Figure 7A,F,G). Ant II with 18 chaetae, two ventral and 16 dorsal; distal row with seven chaetae and one bothriotrichum-like sens ventrally; proximal row with three chaetae (Figure 7A). Ant I with seven chaetae, six dorsal, one smaller, as well as one ventral chaeta (Figure 7A). Head length of holotype as 0.027 mm, in males 0.026–0.027 mm, and females 0.032–0.034 mm. Eyes 8 + 8, A–B, E–F and H larger, D smaller, others subequal; two subequal interocular chaetae present (Figure 5A, Figure 8A and Figure 9A). Labrum distally with two inner papillae and one W-shaped crest and four (**a1–2**), five (**m0–2**) and five (**p0–2**) chaetae, **a2** and **p0–1** smaller, **p2** larger, others subequal; prelabral (**pl**) chaetotaxy with 3 + 3 subequal chaetae (Figure 8A and Figure 9A,B). Labium with one minute and five normal and smooth lpc (Figure 8C). Labial palp with six main papillae (H, A–E), formula of guard appendages as: H(2), A(0), B(4), C(0), D(5), E(5) + l.p. finger-shaped, smooth and not reaching the a.a. base (Figure 8D). Mandibles typical, right with five and left with four incisive teeth, without any other modifications. Maxillae with three outer teeth and six lamellae, two larger in leaf-shape, two elongated and capitate, and two unequal and pin-shaped (Figure 9C). Maxillary outer lobe with t.a. and b.c. subequal in length; sublobal plate (sl.p.) with one appendage and oral fold with two chaetae, all smooth (Figure 9D). Basomedian and basolateral labial fields respectively with four and five chaetae, three slightly smaller and two larger, others subequal (Figure 8E). Postlabial chaetotaxy with four transversal chaetae of a line (Figure 8E).

Male’s head chaetotaxy (Figure 5A and Figure 8A): Clypeal **a**–**f** lines respectively with 7–8, 1 + 5–6, 6,7,6,0–1 + 6 chaetae plus one extra chaeta between **b** and **c** lines and another one between **d** and **e** lines, one larger chaeta of **e** and **f** lines each, others subequal; interantennal area with **α**, **β** and **γ** lines with 1–2, 2, 2–3 chaetae, respectively; **α** outer chaeta larger, **γ** central chaetae modified, inferior **γ** chaeta robust, short and apically wide with a rounded apex, superior **γ** chaeta spine-like apically hooked (Figure 5A and Figure 8A–C); frontal area with **A**–**E** lines, plus 1–2 extra lines, with 2, 2, 2 (present or absent), 2, 1 + 1–2, 1 + 2 and 3 (unpaired absent) chaetae, **AB**–**D** lines with 5–9 spine-like chaetae, and 0–4 as spines (one in **BCD** lines plus another between **AB** and **B** lines), holotype with four spines.

Female’s head chaetotaxy (Figure 9A): Clypeal **a**–**f** lines respectively with 8,1 + 7, 0–1 + 7,5,6,0–1 + 5–6 chaetae plus one extra chaeta between **b** and **c** lines and another one between **d** and **e** lines, all subequal in length; interantennal area with **α**, **β** and **γ** lines with two, two and three subequal chaetae; frontal area with **A**–**E** lines (1 extra line, **AB**) with 2, 1–2, 2, 1 + 2, 1 + 3 and 1 + 3 chaetae, all subequal in length, except for 1 **B** and **C** lines as spine-like chaetae; **A**–**B** lines sometimes misaligned in both sexes.

Male’s large abdomen (Figure 5B and Figure 10A,B): Th II with two **m** chaetae, Th III **m** and **p** with two and 6–7 chaetae, respectively, 1–4 as spines or spine-like chaetae. Abd I **a**, **m** and **p** with five, two and two chaetae (one spine in each line), respectively. Abd II **a**, **m** and **p** with 7–8, eight and nine chaetae, respectively; **a** and **m** with three spines and one spine-like chaeta, **p** with 3–4 spines and 1–2 spine-like chaetae, each line with **ABC** bothriotricha aligned transversally. Abd III **a**, **m** and **p** with eight, seven and six chaetae, respectively; **a** with three spine-like chaetae, **m** and **p** respectively with one and 0–1 spine, and one and 1–2 spine-like chaetae. Abd IV **a**, **m** and **p** with five, six and four chaetae, respectively; **a** with three and **b** and **c** with one spine-like chaetae, respectively, **m** with one small bothriotrichum-like sens on the inferior region. Posterior region dorsally with 2–3, three and two chaetae and one oval organ. Furcula basis with 15 chaetae with different sizes but with same homology in both sexes (Figure 10 and Figure 11).

Female’s large abdomen (Figure 11A,B): Pattern of Th and Abd **amp** lines similar to the male’s chaetotaxy, but with smooth chaetae (devoid of spines and spine-like chaetae). Posterior region dorsally with one, three and two chaetae plus one unpaired chaeta and one oval organ. Large abdomen posteriorly sometimes laterally projected, with a subtriangular shape seen dorsally in both sexes (e.g., Figure 4C). Male’s small abdomen (Figure 10A and Figure 12): Abd V **a**, **m** and **p** respectively with 3–4, five and two chaetae, **a** with one smaller chaeta present or absent, **a** and **m** with **D** and **E** bothriotricha, respectively. Abd VI superior lobe with 1 + 3 (**as1–4**), 1 + 2 (**ams1–3**), 1 + 2 (**ms1–3**), 2 (**mps1–2**) and 1 + 1 (**ps1–2**) chaetae of different sizes, plus one oval organ. Inferior lobes with two (**aai1–2**), five (**ai1–3, ai5–6**), five (**mi1–5**), three (**pi1–3**) and one oval organ (**ami1**) between **ai2** and **ai3**; chaetae **ai1**, **mi2** and **mi4** longer than the others.

**Figure 6:**
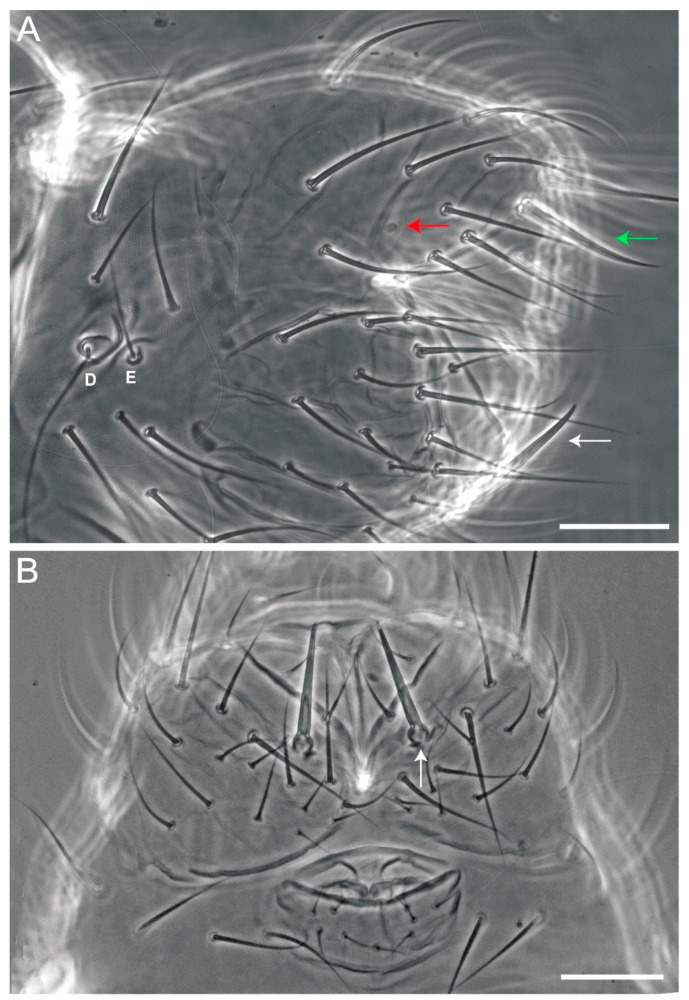
*Arlesminthurus caatinguensis* sp. nov. female structures. (**A**) Small abdomen (lateral view); D and E represent the bothriotricha, the red arrow an oval organ, the green arrow a robust extra spine-like chaeta on the superior lobe of the Abd VI, and the white arrow the subanal appendage (**mi5**) on the inferior lobe of the Abd VI. (**B**) Small abdomen and genital plate (ventral view); white arrow shows the subanal appendage (**mi5**). Scale bars: 0.04 mm.

Female’s small abdomen (Figure 6, Figure 11A and Figure 13): Pattern of Abd V **amp** lines as in the male’s small abdomen. Abd VI superior lobe with 1 + 3 (**as1–4**), 1 + 2 (**ams1–3**), 1 + 2 (**ms1–3**), three (**mps1–3**) and 1 + 1 (**ps1–2**) chaetae of different sizes, plus one robust spine-like chaeta (**?**) between **mps** and **ps** lines, and one oval organ. Inferior lobes with four (**aai1–4**), seven (**ai1–7**), seven (**mi1–7**), three (**mpi1–3**), three (**pi1–3**) and one oval organ (**ami1**) between **ai** and **mi** lines; chaetae **mpi3** and **mi4** longer than the others, subanal appendage (**mi5**) typical, spine-like, smooth and acuminate and on a round tubercle. Small abdomen projected dorso-posteriorly as a finger-shaped structure (Figure 4), ratio small:large abdomen = 1:4.6 in holotype, in males as 1:4.38–4.60 (*n* = 3), in females as 1:4.16–4.71 (*n* = 3).

Genital plate: Males with one pregenital, 4–5 circumgenital and three eugenital chaetae (Figure 12A,D). Females with one unpaired and 4–5 circumgenital on anterior valve, pregenital and eugenital chaetae absent (Figure 6B and Figure 13A,B,D).

Legs (Figure 14, Figure 15 and Figure 16): Legs ratio as I:II:II = 1:1.04:1.40 in holotype. Coxae (epicoxae, subcoxae and coxae) with 0/1/1(I), 1/1/3(II), and 1/1/4 (III) chaetae, respectively. Trochanters I–III with four, six and six chaetae (one on posterior side in all of them), respectively. Femurs I–III with 15, 17 and 17 chaetae, respectively. Tibiotarsi I–III with 52, 52 and 56 chaetae, respectively, inner side with three spine-like chaetae (i) on III–V whorls; distal whorl (I) with 10 chaetae (**e, ae, a, ai, Ja, Jp, pi, p, pe, K**), I–II with three (**K, pe, p**) and III with two (**K, pe**) clavate tenent hairs, respectively. Pretarsus I–III with one small anterior chaeta. Unguiculi I–III trilamellate (**ai, ae, pe**), **pi** lamella absent, as well as tunica and pseudonychia; inner side with one small unpaired tooth on the basal half. Unguiculus I thin and with an apical filament pointed and reaching the distal two thirds of unguis; II–III wide, leaf-shaped, II reaching the basal half of unguis, and III up to the distal one fifth. 

Abdominal appendages (Figure 17): Collophore corpus without chaetae, with one anterior chaeta on each lateral flap and one pair of long warty sacs (Figure 10A and Figure 17A). Tenaculum with three teeth on each ramus, **tb** tube-shaped, **db** wide and rounded, **da + dm** finger-shaped and laterally projected, **la** distally with an apical paired and one unpaired subapical chaeta (Figure 17B). Furcula subequal to the large abdomen length (Figure 4B and Figure 17C); ratio mucro:dens:manubrium = 1:2.94:2.47 in holotype, in males as 1:2.94–3.06:2.47–2.59, and females as 1:2.81–2.91:2.54–2.69. Manubrium with nine dorsal chaetae, one larger and one smaller on latero-distal region, others subequal; ventrally devoid of chaetae. Dens with 33 chaetae, eight on dorsal line (**pi, pe** and six of **p** whorl), eight on outer line, somewhat elongated, eight on inner line, of which five median are clearly elongated, and nine ventral chaetae of formula 3(**ae, ai, e**),3,1,1…1(**a**) chaeta. Mucro with a ventral crest abruptly pointed distally and two lamellae (inner and outer), each lamella with two invaginations (one median and one subdistal), median invagination more evident (Figure 17C).

Etymology: Refers to the Caatinga biome where the new species was found.

Remarks: *Arlesminthurus caatinguensis* sp. nov. resembles *A. salinensis* (Arlé, 1971) [12] from the Pará state, Brazil, and *A. aueti* (Arlé, 1961) [11], of which the type locality is the “Tatuari” River, currently known as “Tuatuari” River, situated in the Indigenous Park of “Xingu”, near Gaúcha do Norte municipalities, the Amazon Biome of the Mato Grosso State, Brazil. These species are similar based on the absence of sexual dimorphism in color patterns, the dens inner row with seven larger chaetae, and wide mucro with invaginations on the outer side (Table 1). However, *A. caatinguensis* sp. nov. is more similar to *A. aueti* based on the head with a median longitudinal strip, the large abdomen anteriorly with two lateral spots and one longitudinal strip depigmented, the male’s head with interocular spines (absent in *A. salinensis*) and the **γ** inferior short and robust frontal chaeta (normal and hooked at the apex in *A. salinensis*) and the mucro with two invaginations on the outer side (four in *A. salinensis*). However, *A. caatinguensis* sp. nov. differs from *A. aueti* based on the Ant IV with eight subsegments (6–7 in *A. aueti*), the male’s **γ** superior frontal chaeta, which is apically hooked, and the **f** line unpaired chaeta, which is subequal to the others (**γ** superior chaeta is swollen and **f** unpaired chaeta is larger in *A. aueti*), the unguis inner tooth (absent in *A. aueti*) and the mucro inner edge with two invaginations (irregular in *A. aueti*). In addition to these characteristics, the new species also differs based on the dens ventral formula with 3,3,1,1…1 chaeta, whereas in *A. aueti* it is apparently 1,3,1,1…1 (see Arlé [12], figure 24).

In *A. salinensis* the color pattern is diffuse on the head and the occipital region and the body is depigmented, whereas in *A. caatinguensis* sp. nov. there is a distinct color pattern, as previous described and illustrated in Figure 4. *Arlesminthurus caatinguensis* sp. nov. also differs from the first species based on the unguis inner tooth, which is present and well developed (vestigial in *A. salinensis*), and the mucro with two invaginations on the inner side (smooth in *A. salinensis*).

On the small abdomen of the female of *A. caatinguensis* sp. nov. there is a robust spine-like chaeta (**?**) between **mps1** and **mps2** chaetae (Figure 6A and Figure 13A,B,D), without a clear homology (following Betsch 1997 [31]), which has never been reported in any species of *Arlesminthurus* before [4,11,12,13]. This feature may be an autapomorphy of the new species; however, it should be further investigated in other species of the genus, especially the Brazilian ones, which lack details on the small abdomen chaetotaxy. Other features like the head and large abdomen spines of males, the presence of an oval organ on the dorso-posterior region of the large abdomen and on the superior and inferior lobes of the small abdomen (Figure 6A) may be shared, at least, among some of the Brazilian species and must be better studied in previously described taxa. Further comparisons among *Arlesminthurus* species are presented in Table 1.

With the description of the new species, we intend to provide a detailed map of chaetotaxic homologies and other morphological aspects of *Arlesminthurus*, to provide further foundations for future species descriptions and comparisons within the genus and among the Bourletiellidae.

### 3.3. *Identification Key and Distribution of* Arlesminthurus *Species*

**Figure 7:**
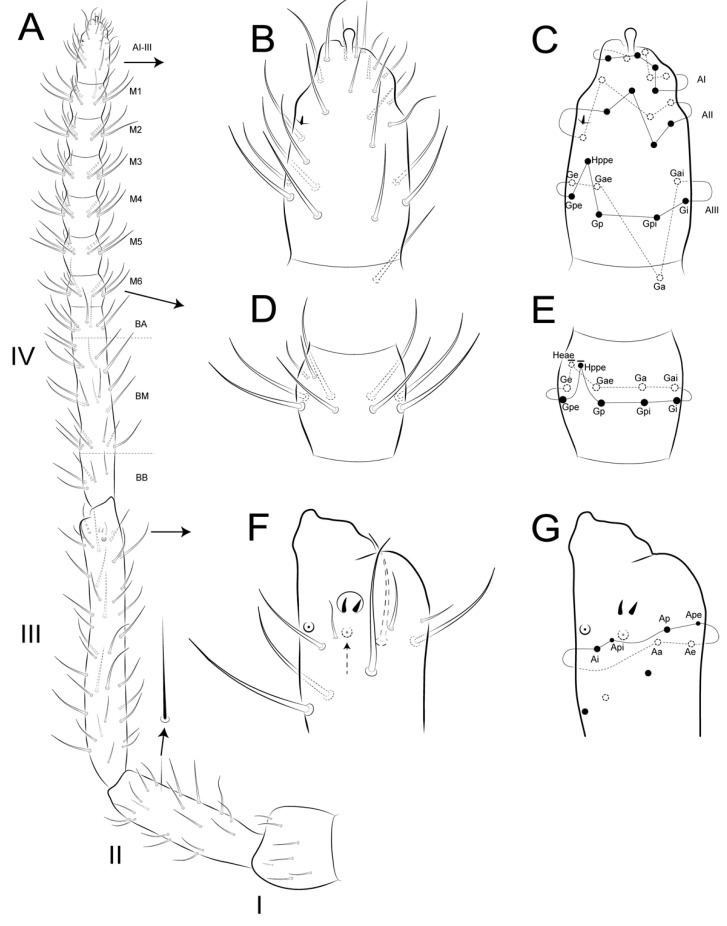
*Arlesminthurus caatinguensis* sp. nov., male antenna: (**A**) complete chaetotaxy of left Ant I–IV (dorsal view); (**B**,**C**) Ant IV apical subsegment (AI–III) (dorsal view): (**B**) chaetotaxy as observed, (**C**) schematic representation; (**D**,**E**) Ant IV subsegments (M1–6) (dorsal view): (**D**) chaetotaxy as observed, (**E**) schematic representation; (**F**,**G**) Ant III apex (ventral view): (**F**) chaetotaxy as observed, dashed arrow indicates a dorsal organ-like microsensilla absent in the females, (**G**) schematic representation.

**Figure 8:**
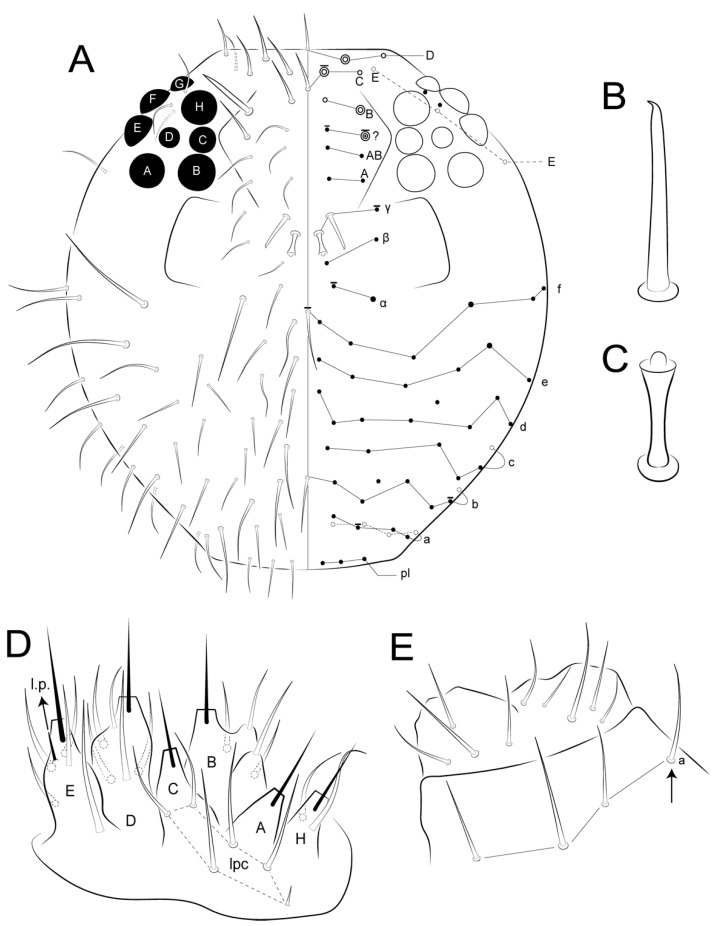
*Arlesminthurus caatinguensis* sp. nov., male: (**A**) complete head dorsal chaetotaxy (prelabral, clypeal, frontal, interocular and occipital regions); the left side shows the chaetae as observed and the right side a schematic representation; (**B**,**C**) frontal chaetae modified on the **γ** line; (**B**) superior chaeta, (**C**) inferior chaeta; (**D**) labial papillae (A–E, and H), and **lpc** (left side); (**E**) basomedian and basolateral labial fields and postlabial chaetotaxy (right side); arrow indicates ventral chaetae of the **a** line.

1. Color patterns sexually dimorphic; dens inner row with six chaetae slightly larger than the others … 2

- Both sexes with the same color pattern (Figure 4); dens inner row with seven chaetae clearly larger than the others (Figure 17C) … 3

2. Male head with one postocullar and one lateral band of pigment, females with two small postocular spots; large abdomen with an evident W-shaped spot in males; Ant IV with 9–11 subsegments; male **γ** frontal chaetae apically fringed; unguis III pseudonychia present; mucro normal with smooth edges … *A. franzkafkai* Palacios-Vargas and Cabrera, 2015 [13] (Nicaragua)

- Males with two longitudinal bands on lateral body, female head with one interantennal spot and with the large abdomen with three depigmented spots; Ant IV with eight subsegments; male **γ** frontal chaetae apically swollen; unguis III pseudonychia absent; mucro wide with irregular edges … *A. richardsi* (Arlé, 1971) [12] (Brazil)

3. Head color diffuse, with occipital region, as well as the body, depigmented; male head with inferior frontal chaetae (**γ**) apically hooked; mucro outer edge with four invaginations … *A. salinensis* (Arlé, 1971) [12] (Brazil)

- Head with one longitudinal strip of pigment, body with two lateral spots and one longitudinal depigmented strip on the anterior region (Figure 4A,C); male head with inferior frontal chaetae (**γ**) short and robust (Figure 5A, and Figure 8A,C); mucro outer edge with two invaginations (Figure 17C) … 4

4. Ant IV with 6–7 subsegments; male with superior frontal chaetae (**γ**) apically swollen and **f** line unpaired chaeta larger than the others; unguis inner tooth absent; mucro inner edge irregular … *A. aueti* (Arlé, 1961) [11] (Brazil)

- Ant IV with eight subsegments (Figure 7A); male with superior frontal chaetae (**γ**) apically hooked and **f** line unpaired chaeta subequal to the others (Figure 5A and Figure 8A,B); unguis inner tooth present (Figure 16B); mucro inner edge with two invaginations (Figure 17C) … *A. caatinguensis* sp. nov. (Brazil)

### 3.4. *Taxonomic Summary of* Calvatomina *and Genus Diagnosis*

Order Symphypleona Börner, 1901 [32] sensu Bretfeld, 1986 [3]

Suborder Appendiciphora Bretfeld, 1986 [3]

Superfamily Dicyrtomoidea Bretfeld 1994 [1]

Family Dicyrtomidae Börner, 1906 [19]

Subfamily Dicyrtominae Richards, 1968 [35] sensu Bretfeld 1999 [4]

Genus *Calvatomina* Yosii, 1966 [8] sensu Betsch, 1980 [36]

Genus diagnosis: Frontal head and anterior region of the large abdomen with only small chaetae, without long chaetae or robust spines. Large abdomen posteriorly with spines, bothriotrichum **A** present, **D** absent in adults. Abdominal protuberance absent. Neosminthuroid chaetae present on parafurcal area. Small abdomen mostly with blunt acanthoid chaetae, with up to three bothriotricha-like sens. Tenaculum with 3 + 3 or 4 + 4 teeth. Ungues with tunica. Dental chaetae smooth or slightly serrate (adapted from [4,8,18,36,37]).

Type species: *Dicyrtomina* (*Calvatomina*) *cruciata* Yosii, 1966 [8].

Remarks on the genus: We made few modifications to the diagnosis provided by Betsch [36] and Bretfeld [4]. Among the *Calvatomina* species, at least few of them, like *C. guyanensis* Nayrolles and Betsch, 1995 [18], *C. lawrencei* (Yosii, 1969) [37] and *C. trivandrana* (Prabhoo, 1971) [38], have a tridentate tenaculum. Furthermore, some species, like *C. bougainvilleae* (Yosii, 1960) [39], *C. guyanensis, C. modesta* (Yosii, 1969) [37] and the new one herein described, lack clear modified chaetae on the posterior distal tibiotarsus.

Remarks on the Neotropical fauna: *Calvatomina rufescens* (Reuter, 1890) [40] is one of the most puzzling species of the genus. It was originally described by Reuter based on specimens collected from a greenhouse in Helsinki, Finland. Subsequently, it was redescribed by Hüther [41] after the analysis of its type material. Hüther also suggested that the species was introduced, as it shows many resemblances with other tropical taxa and it was only collected from greenhouses [4,16,41]. *Calvatomina rufescens* has been considered widespread, but most records outside of its type locality are doubtful due to incongruences in the chaetotaxy of the females’ small abdomen [2,4]. However, this is not the case for the specimens from Colombia and Puerto Rico [16,17]. Although the drawings of Mari-Mutt do not represent chaeta **as1**(**M**) as blunt acanthoid, in his notes the author explains that **M** is polymorphic in the Colombian specimens, and it can be a regular pointed or a long blunt acanthoid chaeta, as well as **as3**(**N’**), which can be a thick or a regular pointed chaeta (Mari-Mutt [16] pp. 377–378, figures 64 and 65). The same polymorphism of the **M** chaeta was recorded to Puerto Rican specimens by Soto-Adames ([17], pp. 64–65, figure 20). Considering such polymorphism, the only clear differences among Mari-Mutt, Soto-Adames and Hüther’s drawings are the absence of the short regular chaetae **mps’**(**G**) and **ami1**(**T**) in the latter’s representations, which could be easily overseen due to their positions. To endorse this point of view, Hüther also did not represent chaetae **ps1–2**, which are primary elements seen in most if not all Symphypleona [31]. Hüther’s redescription of *C. rufescens* also matches the notes of Mari-Mutt in key features like color pattern, empodial complex morphology, chaetotaxy of the parafurcal area, collophore with one chaeta on each side, tenaculum with two chaetae, manubrium with 10 chaetae and dens ventral chaetae formula from the apex to the basis as 3,2,1,1,0,0,1, with the proximal chaeta smaller and far apart from the others. Because of this, at this time and with the current knowledge of the species morphology, we believe the records of Neotropical *C. rufescens* from Colombia and Puerto Rico are valid, since they fit the redescription provided by Hüther [41]. Even so, this conclusion does not exclude the possibility that *C. rufescens* could represent a species-complex, due to its widespread distribution and since further data not represented by the previous authors may have obscured closely related taxa.

*Calvatomina rufescens* var. *discolor* (Schött, 1902) [42] is a similar case to *C. rufescens*. This variation of the late species was described in Sweden based on material also collected from greenhouses [16,42]. Schött presented a short description lacking chaetotaxic data, but remarked that his specimens’ color pattern was characteristic and distinct from that of *C. rufescens* described by Reuter [40]. *Calvatomina rufescens* var. *discolor* was not revised by Hüther, but it was considered by the author to be “of the same species” in a short note provided in his revision, which lacks any comparative data or further explanation (Hüther [41], pp. 50–51), and it was disregarded by important revisions like Bretfeld [4] and Fjellberg [43]. However, Mari-Mutt [16] proposed a new status to *C. rufescens* var. *discolor* as a full species based on material collected from Colombia. His specimens presented the same J-shaped band of purple-violet pigment laterally on the large abdomen described by Schött and were similar to the Colombian specimens of *C. rufescens*. Even so, a similar color pattern was also described to *C. christianseni* (Delamare-Debouteville and Massoud, 1964) [15] in Suriname (see Delamare-Debouteville and Massoud [15], p. 78, figure 40B) and is similar to *C. nymphascopula* Soto-Adames, 1988 [17] from Puerto Rico (see Soto-Adames [17], p. 66, figure 22). Since there are no further useful data on the morphology of *C. discolor* from Sweden, at this time we cannot confirm if the Colombian species matches the Swedish one, nor can we refute this hypothesis. We thus consider *Calvatomina discolor* as a *species inquirenda*, and its type material, if available, must be redescribed in order to clearly circumscribe its identity. Nevertheless, Mari-Mutt’s description of *C. discolor* is detailed and represents a distinct Neotropical species, so we provisionally propose *C. discolor* sensu Mari-Mutt [16] to stand for it until further data on the material from the type locality are presented.

### 3.5. Calvatomina gladiata *sp. nov. Medeiros, Bellini and Cipola*

Figure 1, Figure 18Figure 19, Figure 20, Figure 21, Figure 22 and Figure 23.

Type material: Holotype female in slide (CC/UFRN): Brazil, Rio Grande do Norte state, Natal municipality, “Mata dos Saguis”, Biosciences Center of the Federal University of Rio Grande do Norte (05°50′34.30″ S, 35°12′04.63″ W), 6 April 2017, entomological aspirator, GS Medeiros and BC Bellini coll. Sixteen paratypes on slides: three females, four males and one juvenile, same data as holotype; one female and five males, same data as holotype except 03 June 2015, NMC Santos and PGC Souza; one female, as above, except 11 March 2015; one female and one juvenile, same data as holotype, except 09 March 2020, GS Medeiros and AC Batista. All material deposited at CC/UFRN, except one male and one female at INPA (INPA-CLL 000118).

Diagnosis: Specimens mostly brownish, with a light sword image on dorso-anterior large abdomen (Figure 19). Ant III with 10, Ant II with two cup sensilla, Ant I with seven regular subequal chaetae (Figure 20A–C). Head clypeal area with 4–5 unpaired central chaetae and two, one, two, one cup sensilla on lines **b, c, e** and **f**, respectively, line **f** with one acanthoid chaeta laterally to antennal basis (Figure 20D,E). Sublobal plate with one chaeta-like appendage (Figure 20K). Labial papillae with five proximal chaetae (Figure 20L). Epicoxae II–III with one acanthoid chaeta each; femurs II–III with one oval organ each, tibiotarsi I–III with four each; tibiotarsi I–III with five cup sensilla each (Figure 21A–C). Collophore with two chaetae on each side, tenaculum with two chaetae on each side. Manubrium with nine dorsal chaetae on each side (Figure 21G). Dens dorsally with 25 chaetae; dens ventral formula from apex to the basis as: 4,2,1,1,0,0,1 (Figure 21H,I). Parafurcal area (furcula basis) with 4–5 neosminthuroid chaetae plus two cup sensilla (Figure 22). Small abdomen with three blunt acanthoid chaetae on the dorsal anal valve (**ms1, ms2** and **ms4** on females, **ms1, ms2** and **mps1** on males), plus three cup sensilla (**as4, as5** and **a1** on both genders), **mi1** on ventral anal valves of females also as a blunt acanthoid chaeta (Figure 23).

Description: Body (head + trunk) length of type series ranging between 0.79 and 1.2 mm, males average 0.87 mm, females average 0.82 mm, holotype with 1.2 mm. Habitus typical of the genus. Body and appendages with different types of chaetae as presented in Figure 18. Body color in ethanol mottled brown, darker and more uniform on antennae, lateral head and trunk, femurs and tibiotarsi, without pigments on the surrounding region of the eyes and furcula; dorsally lighter and spottier, with an inverted sword image on anterior region of the large abdomen (Figure 19). Body chaetae smooth and acuminate, with the exception of few chaetae on small anal valves.

Head (Figure 19 and Figure 20): Antennae shorter than body, with 0.66 mm in the holotype (Figure 19). Holotype antennal segments ratio of Ant I:II:III:IV as 1:3:11.8:2.2. Ant IV with about 54 regular chaetae plus a small subapical “mushroom-like” organ (Figure 20A). Ant III with a constriction in the basal 2/5, almost subsegmented, with 58 regular chaetae plus 10 cup sensilla, apical organ typical with two sense rods inside a single invagination, accessory microsensillum present (Figure 20A,B). Ant. II with 20 regular chaetae plus two cup sensilla (Figure 20C). Ant I with seven regular chaetae, subequal in size (Figure 20C). Head length (eyes to mouth) of holotype 312 µm. Clypeal area **a–f** lines with 8/7(+1)/4/4(+1)/3(+1)/5(+1) chaetae respectively, two extra chaetae with unclear homologies (circled), one of them unpaired; **b, c, e** and **f** lines with two, one, two, one cup sensilla, respectively; **f** line with one lateral acanthoid chaeta, near to the antennal basis (Figure 20D–F). Interantennal area **α, β** and **γ** lines with two, two, one short chaetae, respectively, three small pseudopore-like organs present nearby to the **α** line; frontal area with only **A, D** and **E** lines, with two, three, three short chaetae, respectively; **E** line with one small cup sensillum behind the eyes; eyes 8 + 8, with two interocular chaetae (Figure 20D). Distal margin of the clypeus with three prelabral chaetae, labral chaetotaxy with 2(+1) **p**, 2 (+1) **m** and two **a** chaetae, **a2** as an almost blunt chaeta (somehow spiniform), **m1** larger than all other labral chaetae; four labral crests present, the two internal apically subdivided; labrum without clear formed papillae (Figure 20D,G,H). Mandibles asymmetrical with 5–6 apical incisive teeth (Figure 20I); maxillae typical with six lamellae (Figure 20J); maxillary outer lobe with apical chaeta about three times longer than the basal one, sublobal plate with one inner chaeta-like appendage plus an outer short cuticular projection (Figure 20K). Ventral groove with two surrounding chaetae from lines **a** and **b**, labial basomedian field projected laterally with four central (one larger than the others) and three lateral chaetae, basolateral field with two subequal chaetae (Figure 20F). Labial palp with five proximal subequal chaetae, labial papilla formula of guard chaetae as H(2), A(0), B(3), C(0), D(4), E(5) + a blunt finger-shaped lateral process not reaching the papilla apex (Figure 20L,M). 

Large abdomen appendages (Figure 21): Epicoxa, subcoxa and coxa I with zero, one, zero chaetae, respectively; trochanter I with four chaetae; femur I with 11 regular chaetae plus one microsensillum and one cup sensillum; tibiotarsus I with 44 regular chaetae (seven of them in the inner side thicker, proximal **Fpae, Fppe** and **Fse↓** present), plus five cup sensilla (one of them as proximal **Fpe**) and four oval organs (**O4ae, O4pe, O3pe** and **O2pe**), distal whorl with eight chaetae (Figure 21A). Epicoxa, subcoxa and coxa II with one, one, three chaetae, respectively, epicoxa chaeta acanthoid; trochanter II with 4–5 chaetae; femur II with 14 regular chaetae plus one microsensillum, one cup sensillum and one oval organ; tibiotarsus II with 44 regular chaetae (seven of them in the inner side thicker, proximal **Fpae, Fppe** and **Fse↓** present), plus five cup sensilla (one of them as proximal **Fpe**) and four oval organs (**O4ae, O4pe, O3pe** and **O2pe**), distal whorl with eight normal chaetae (Figure 21B). Epicoxa, subcoxa and coxa III with one, one, four chaetae, respectively, epicoxa chaeta acanthoid; trochanter III with six chaetae; femur III with 12 regular chaetae plus one microsensillum, one cup sensillum and one oval organ; tibiotarsus III with 41–44 chaetae (11–12 of them in the inner side thicker, proximal **Fpae, Fppe** and **Fse↓** present), plus five cup sensilla (one of them as proximal **Fpe**) and four oval organs (**O4ae, O4pe, O3pe** and **O2pe**), distal whorl with eight chaetae (Figure 21C). Foot complexes I–III with two pretarsal chaetae each, one anterior and one posterior; ungues without cavity with two unpaired inner teeth subequal in size, lateral lamellae without teeth, pseudonychia present, merged to the dorsal face with two dorsal teeth, the distal one smaller, ending in a subapical projection, with lateral margins irregularly serrate, tunica present at the apex of the ungues (Figure 21D–F). Unguiculi trilamellate without the postero-internal lamella, with two teeth on antero-internal lamella, proximal one enlarged; unguiculi with filament on the posterior lamella, nearside the apical tooth, unguiculus I filament surpassing the unguis apex, shorter on unguiculi II and III. Collophore corpus with one posterior chaeta on each side, plus one distal chaeta on each lateral flap, with a pair of warty sacs. Tenaculum with three teeth on each ramus plus the basal tubercle, with two chaetae on each side of the corpus. Furcal size length in holotype as: manubrium = 74 µm; dens = 249 µm; and mucro = 79 µm. Manubrium with nine dorsal chaetae on each side (Figure 21G); dens dorsally (posteriorly) with 25 chaetae on the dorsal, inner and outer lines of chaetae combined, one proximal longer than others (Figure 21H); dens ventrally (anteriorly) with nine chaetae, with the following formula from the apex to the basis: 4,2,1,1,0,0,1 (Figure 21I); mucro with narrow apex, with both edges serrated, with about 20 teeth on each edge (Figure 21H,I). Ratio mucro: dens: manubrium in holotype 1:3.4:1.07.

Trunk (large and small abdomens) (Figure 22 and Figure 23): Trunk length of holotype 1.08 mm. Large abdomen (Figure 22): thorax continuous with abdomen, without any visible segmentation or constrictions. Chaetotaxy similar between males and females, Th II with one **a** and three **m** chaetae; Th III with one **a** and two **m** chaetae; Abd I with three **a** chaetae; bothriotricha **A, B** and **C** present and misaligned, **A** posteriorly to **B–C**, on a large papilla with a cup sensillum, **B** and **C** with 4 accessory chaetae; 0–1 cup sensillum posterior to the bothriotricha, dorso-posterior region with about 15 spine-like chaetae, none unpaired (Figure 22A). Parafurcal area (furcula basis) with 4–5 neosminthuroid chaetae, two cup sensilla and four regular chaetae (Figure 22A,B). Small abdomen of the female (Figure 23A,B): dorsal anal valve with **as1–5, ms1–4, mps’, mps1–4 and ps1–2** chaetae, **as1, ms1** and **ps1** unpaired; **as4–5** as cup sensilla, **ms1–2** and **ms4** as blunt acanthoid chaetae, **ms3** as a **S** chaeta (bothr-sens); ventral anal valves each with **aai1, ai1–6, ami1–6, mi1–5, mpi1–2** and **pi1–3** chaetae, **ami4** as an oval organ present or absent; **ai1** as a cup sensillum, **ai2** and **mi2** as **S** chaetae (bothr-sens), **ami2** and **ami4** as oval organs (Figure 23E), **mi1** as a blunt acanthoid chaeta, **mi5** as the subanal appendage curved toward the anus opening, smooth, blunt and thick. Small abdomen of male (Figure 23C,D): dorsal anal valve with **as1–5, ams2, ms1–4, mps1**, **mps3** and **ps1–2** chaetae, **as1, ms1** and **ps1** unpaired, **as4–5** as cup sensilla, **ms1**–**2** and **mps1** as blunt acanthoid chaetae, **ms3** as bothr-sens; ventral anal valves each with **aai1, ai1–6, ami2, mi1–5** and **pi1–3** chaetae, **ai1** as a cup sensillum, **ai2** and **mi2** as bothr-sens, **ami2** as an oval organ. Another interpretation of the small abdomen chaetotaxy following Nayrolles and Betsch [18] is presented in Figure 23B,D. Genital plates of the female and male unclear.

Etymology: The new species was named after the sword-like image on its dorsum (from Latin gladius = sword).

Habitat: *Calvatomina gladiata* sp. nov. specimens were collected from “Mata dos Saguis”, a small forested area within the Biosciences Center of the Federal University of Rio Grande do Norte campus, Natal, Rio Grande do Norte state, Brazil (Figure 1). Further data on the area is presented in [44]. It is the same type locality of the recent described *Brachystomella nordestina* Souza, Bellini and Weiner, 2018 [44] and *Lepidocyrtinus dapeste* (Santos and Bellini, 2018) [44].

Remarks: Regarding the Neotropical fauna, *Calvatomina gladiata* sp. nov. is superficially similar to the other species of heterogeneous color pattern, with the presence of several cup sensilla on Ant III, II and head, 4–5 unpaired chaetae on clypeal area and 4–5 neosminthuroid chaetae on parafurcal area (Table 2). On the other hand, the new species clearly differs from the Neotropical *Calvatomina* as follows: clypeus with six pairs of cup sensilla (five or eight in the other taxa) and the dorsal dens with dorsal, inner and outer lines summed with 25 chaetae (21–24 in the other species). The new species also differs in ventral dental chaetae formula as 4,2,1,1,0,0,1, with three unpaired basal chaetae, from *C. christianseni* and *C. discolor* sensu Mari-Mutt [16], compared with ventral dental formula as 3–4,2,1,1,1,0,1, with four unpaired basal chaetae, Ant III with 10 cup sensilla (nine in *C. guyanensis*), Ant II with two cup sensilla (three in *C. rufescens* and *C. guyanensis*), the presence of a lateral pair of short acanthoid chaetae on clypeal f line (absent in *C. christianseni*), collophore with two and tenaculum with two chaetae per side, respectively (one and one respectively in *C. discolor* sensu Mari-Mutt [16] and *C. rufescens*) and the manubrium with nine dorsal chaetae (10 in *C. discolor* and *C. rufescens*).

*Calvatomina gladiata* sp. nov. also differs from other Neotropical species in the chaetotaxy of the small abdomen of the female (Table 2). The new species have **as1**(**M**) as regular pointed chaeta (blunt acanthoid in *C. christianseni* and *C. guyanensis*), **as2**(**M’**) as regular pointed chaeta (blunt acanthoid in *C. guyanensis*), **ms1**(**a0**), **ms2**(**N**) and **ms4**(**H**) as blunt acanthoid chaetae (all regular pointed in *C. nymphascopula*, only **ms4**(**H**) as regular pointed in *C. discolor* sensu Mari-Mutt [16]) and **mi1**(**L**) as a blunt acanthoid chaeta (regular pointed in *C. discolor* sensu Mari-Mutt [16] and *C. nymphascopula*). A detailed comparison of the Neotropical *Calvatomina* is presented in Table 2 and in the identification key is presented at the end of the remarks.

The new species fits the *rufescens*-group due to the presence of only three unpaired basal chaetae on the ventral dens and **ms1**(**a0**) as a blunt acanthoid chaeta [4,18,37]. There are three species from the *rufescens*-group recorded in the Neotropical region: *C. guyanensis*, *C. rufescens* and now *C. gladiata* sp. nov. Outside the Neotropical Region, three other taxa from this group are similar to the new species: *C. cruciata*, *C. pallida* (Yosii, 1969) [37] and *C. trivandrana*, all from India, especially in the overall chaetotaxy of the small abdomen of the female with **as1**(**M**), **as2**(**M’**), **as3**(**N’**) and **ami1**(**T**) as small regular pointed chaetae (see Nayrolles and Betsch [18], p. 288, table 2). However, the new species clearly differs from the Indian taxa in: dens ventrally with four chaetae at the apex (vs. 2–3), dorsal dens with 25 chaetae (vs. 23–24), Ant III with 10 and Ant II with two cup sensilla, respectively (eight and three in *C. trivandrana*), clypeus with 4–5 unpaired chaetae (six in *C. cruciata*), collophore with two chaetae per side (one in *C. trivandrana*), and **ms4**(**H**) and **mi1**(**L**) on the females small abdomen as blunt acanthoid chaetae (as regular pointed chaetae in *C. pallida*). Further comparisons among these species are shown in Table 2.

### 3.6. Identification Key and Distribution* of Neotropical Species of Calvatomina

1. Dens ventral chaetae formula from the apex to the basis as 3–4,2,1,1,1,0,1, with four unpaired basal chaetae (*bougainvilleae*-group sensu Yosii, 1969) [38] … 2

- Dens ventral chaetae formula from the apex to the basis as 3–4,2,1,1,0,0,1, with three unpaired basal chaetae … 3

2. Clypeal region lacking the lateral acanthoid chaeta on f line, dens dorsally with 23 chaetae, small abdomen of the female with **as1**(**M**), **ms4**(**H**) and **mi1**(**L**) as blunt acanthoid chaetae … *C. christianseni* (Delamare-Deboutteville and Massoud, 1964) [15] (Suriname)

- Clypeal region with a lateral acanthoid chaeta on f line, dens dorsally with 24 chaetae, small abdomen of the female with **as1**(**M**), **ms4**(**H**) and **mi1**(**L**) as regular pointed chaetae … *C. discolor* (Schött, 1902) [42] sensu Mari-Mutt (1987) [16]** (Sweden, Colombia, Puerto Rico)

3. Small abdomen of the female with **ms1**(**a0**) as a regular pointed chaeta (*formosana*-group sensu Yosii, 1969) [37], chaetae **ms2**(**N**), **ms4**(**H**) and **mi1**(**L**) of the same region and gender also as regular pointed chaetae, dorsal color pattern of the large abdomen with two dark knife-shaped forms … *C. nymphascopula* Soto-Adames, 1988 [17] (Puerto Rico)

- Small abdomen of the female with **ms1**(**a0**) as a blunt acanthoid chaeta (*rufescens*-group sensu Yosii, 1969) [37], chaetae **ms2**(**N**), **ms4**(**H**) and **mi1**(**L**) of the same region and gender also as blunt acanthoid chaetae, dorsal color pattern of the large abdomen otherwise … 4

4. Clypeal region with eight cup sensilla, collophore with one chaeta, tenaculum with one chaeta, manubrium with 10 chaetae … *C. rufescens* (Reuter, 1890) [40] (USA, Finland, Colombia, Cuba, Mexico, Puerto Rico)

- Clypeus with 5–6 cup sensilla, collophore with two chaetae, tenaculum with two chaetae, manubrium with nine chaetae … 5

5. Ant III with nine and Ant II with three cup sensilla, respectively, clypeal region with five cup sensilla, dens dorsally with 24 chaetae, small abdomen of the female with **as1**(**M**) and **as2**(**M’**) as blunt acanthoid chaetae … *C. guyanensis* Nayrolles and Betsch, 1995 [18] (French Guiana)

- Ant III with 10 and Ant II with two cup sensilla, respectively, clypeal region with six cup sensilla, dens dorsally with 25 chaetae, small abdomen of the female with **as1**(**M**) and **as2**(**M’**) as regular pointed chaetae … *C. gladiata* sp. nov. (Brazil)

* Distribution based on the original descriptions and [2,4,45,46,47]. 

** See also the remarks on the Neotropical *Calvatomina* for further data on the identity of this species.

## 4. Discussion

### 4.1. *On the Bothriotrichum-Like Sens of* Arlesminthurus *and the Genus Affinities*

In the new species of *Arlesminthurus* herein described, there is a bothriotrichum-like sens on the inferior side (Abd IV **m** line) of the large abdomen (Figure 5B and Figure 10), which was not described in the other four species of the genus [4,11,12,13]. Apparently, most of the Bourletiellidae genera are devoid of this bothriotrichum-like sens (e.g., [4,36,48,49]). For instance, this structure is absent in the specimens of *Stenognathriopes janssensi* Zeppelini and Silva, 2012 [50] analyzed by us, corroborating the original description of the genus (see Betsch and Lasebikan [51], Figure 1A).

This bothriotrichum-like sens was apparently only observed in *Bourletiella viridescens* Stach, 1920 [52] (see Betsch [36]: p. 98, figure 38E), and this character is quite probably homologous to the S-sens present in the large abdomen of *Bourletiella* Banks, 1899 [53], *Deuterosminthurus* Börner, 1901 [33], *Fasciosminthurus* Gisin, 1960 [54] and *Heterosminthurus* Stach, 1955 [55] [4,36,49,56]. However, its morphology is not detailed and/or determined as diagnostic of such genera, as well as to the overall Symphypleona [4,35,36].

Although no representative phylogenetic study of Bourletiellidae using modern methods has been performed so far, Betsch ([36], p. 188) arbitrarily proposed a clade contemplating the genera *Bourletiella, Deuterosminthurus* and *Heterosminthurus*. This grouping was based on their simple and conical tibiotarsal spines, unguiculi I–III of similar sizes and tenaculum with two teeth per side (see Betsch [36], p. 174). Except for the obliquely truncate tibiotarsal chaetae [4,57,58], these characteristics are also present in *Fasciosminthurus*, which was considered by Betsch as a synonym of *Prorastriopes* Delamare Deboutteville, 1947 [59], and for that reason it was not nominally included in his arbitrary phylogenetic construction (see Betsch [36], p. 175). At that time, three species of *Arlesminthurus* were already described as *Deuterosminthurus* (*D. aueti, D. richardsi* and *D. salinensis*), and more recently *A. franzkafkai* plus *A. caatinguensis* sp. nov. herein described. These five species, and so *Arlesminthurus* as a whole, also fit into the group proposed by Betsch [36], except for the tenaculum morphology, which is variable, with two or three teeth [4].

**Figure 9:**
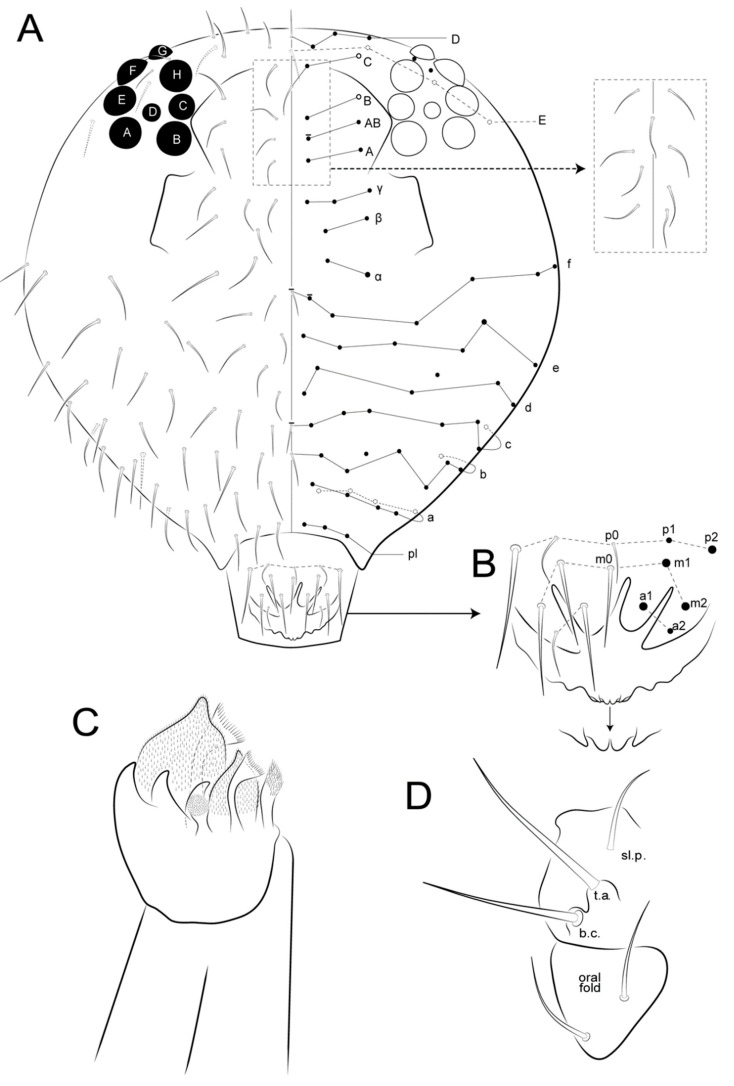
*Arlesminthurus caatinguensis* sp. nov., female: (**A**) complete head dorsal chaetotaxy (prelabral, clypeal, frontal, interocular and occipital); the left side shows the chaetae as observed and the right side shows a schematic representation; arrows show variation interocular chaetae and labral region, respectively; (**B**) labrum, arrow indicates the labral papillae; (**C**) left maxilla apex (ventral side); (**D**) maxillary outer lobe (left side).

**Figure 10:**
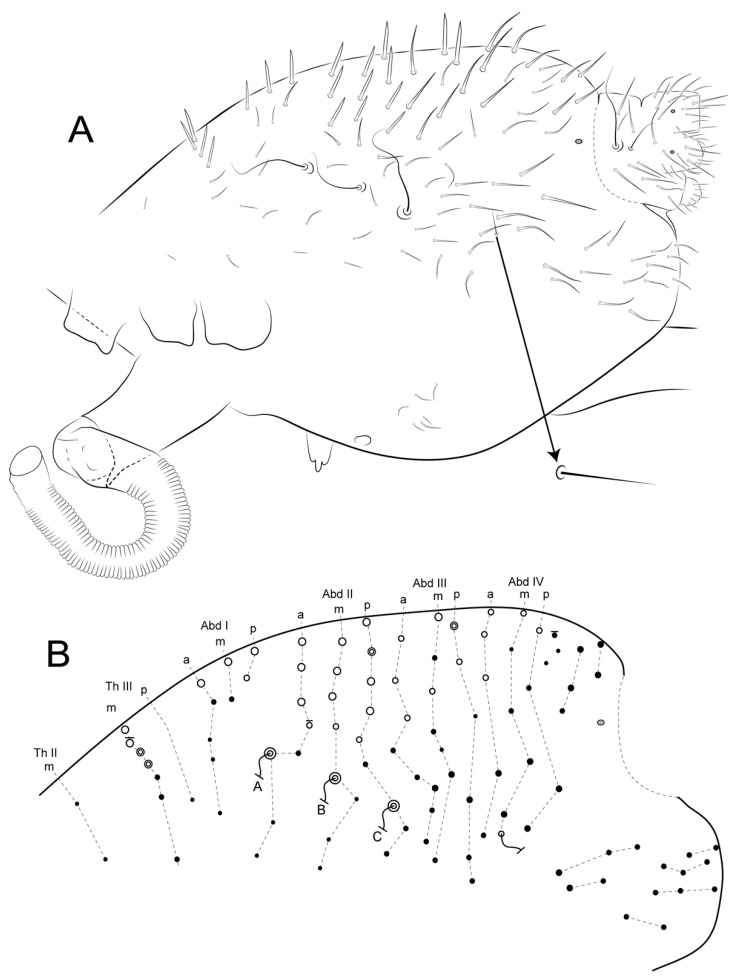
*Arlesminthurus caatinguensis* sp. nov. male, large and small abdomens (lateral view): (**A**) chaetotaxy as observed, arrow indicates extra bothriotrichum; (**B**) schematic representation of the large abdomen.

**Figure 11:**
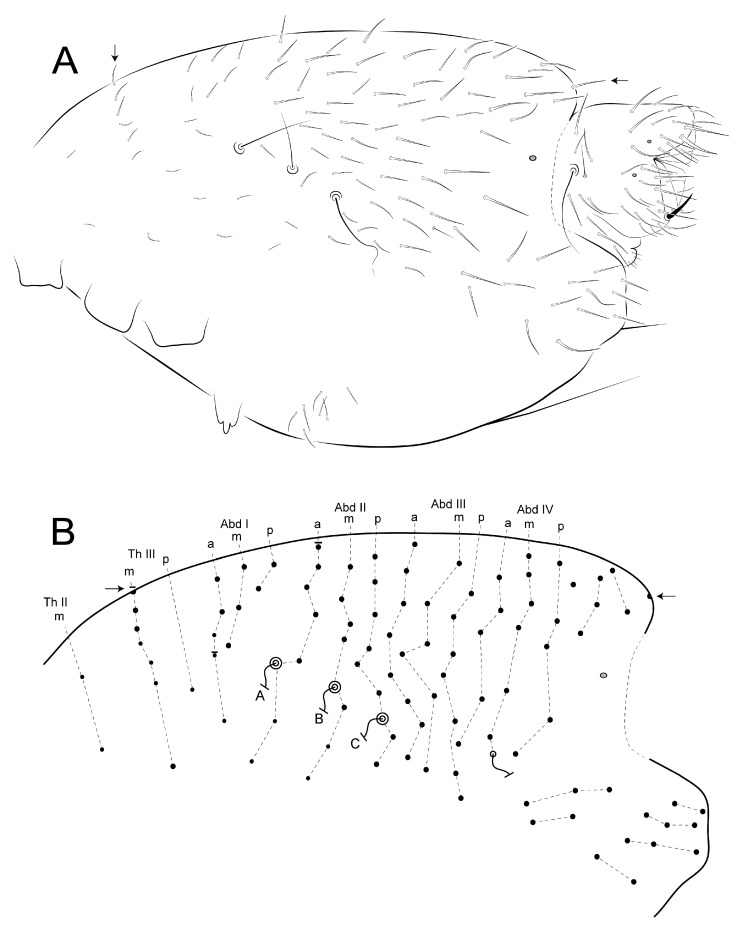
*Arlesminthurus caatinguensis* sp. nov. female, large and small abdomens (lateral view): (**A**) chaetotaxy as observed; (**B**) schematic representation of the large abdomen; arrows on both figures show unpaired chaetae.

**Figure 12:**
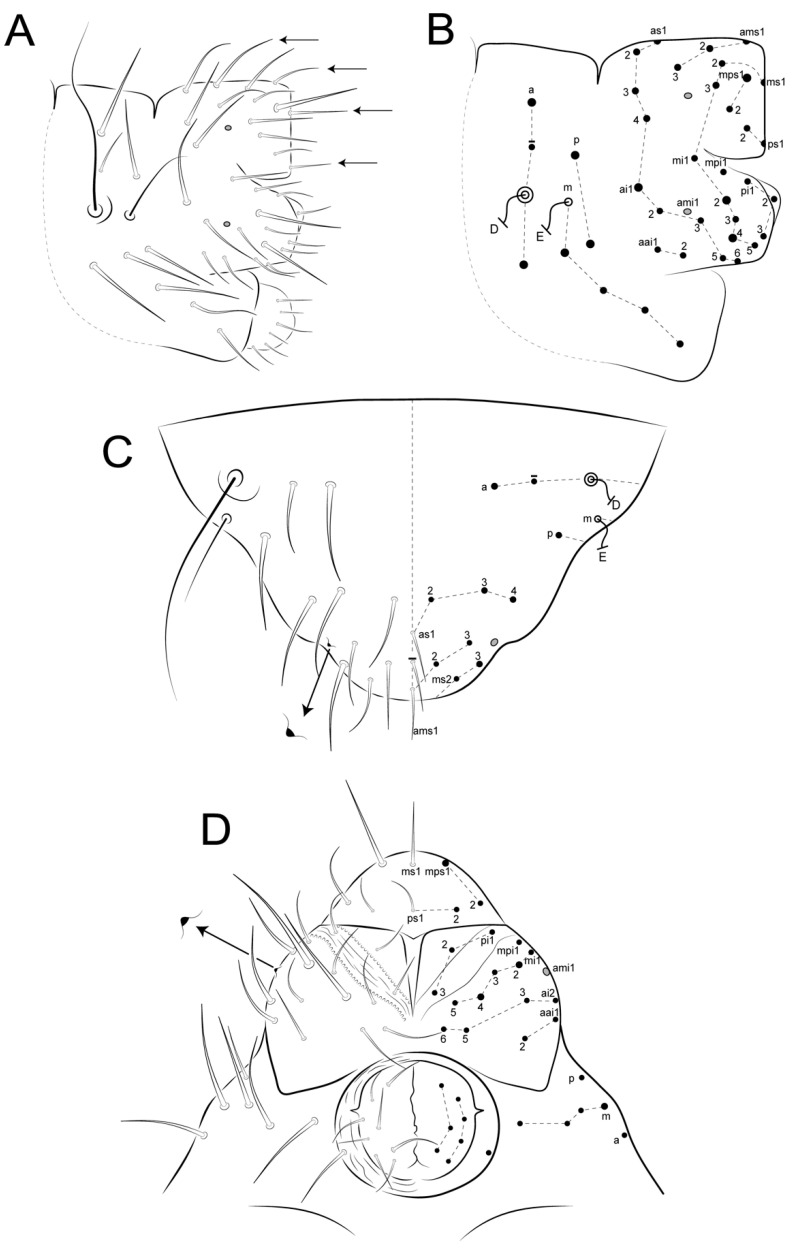
*Arlesminthurus caatinguensis* sp. nov. male small abdomen: (**A**,**B**) lateral view, (**A**) as observed, arrows indicate unpaired chaetae (**as1, ams1, ms1** and **ps1**), (**B**) schematic representation; (**C**) dorsal view; (**D**) ventral view of the small abdomen and the genital plate; arrows in both **C** and **D** figures show the oval organs, the left side shown as observed and on the right side a schematic representation is displayed.

**Figure 13:**
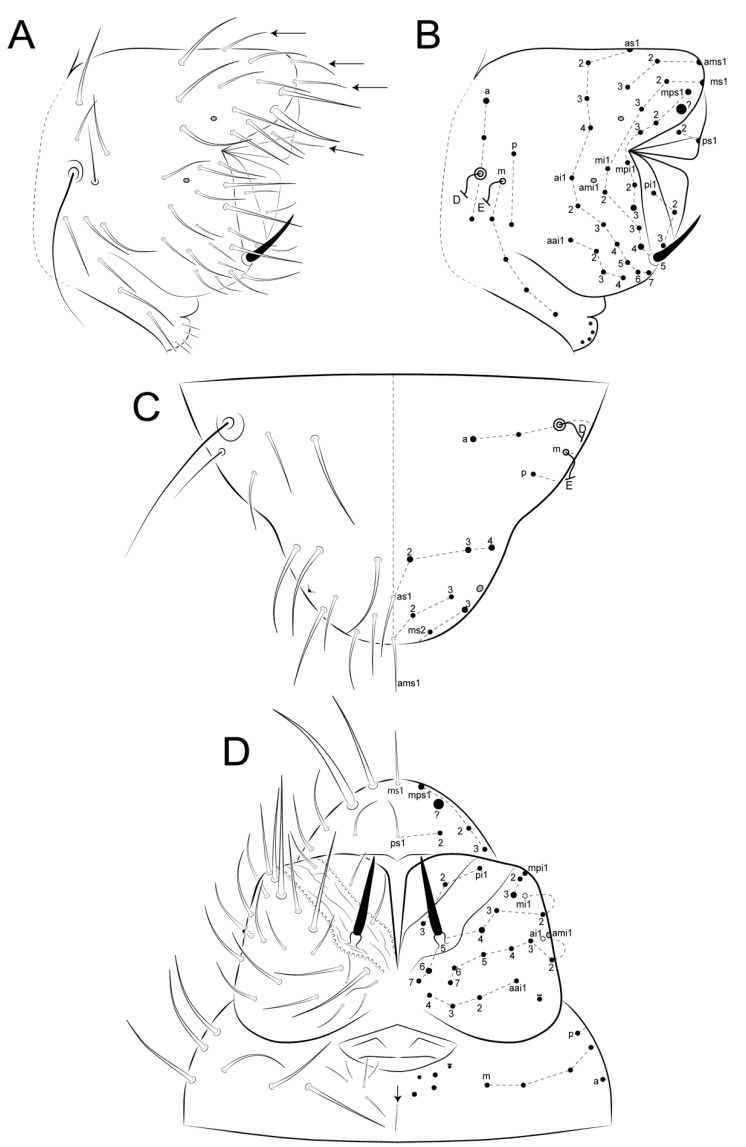
*Arlesminthurus caatinguensis* sp. nov. female small abdomen: (**A**,**B**) lateral view; (**A**) as observed, arrows indicate unpaired chaetae (**as1, ams1, ms1** and **ps1**), (**B**) schematic representation; (**C**) dorsal view; (**D**) ventral view of small abdomen and the genital plate; in both **C** and **D** the left side is shown as observed and the right side is a schematic representation.

**Figure 14:**
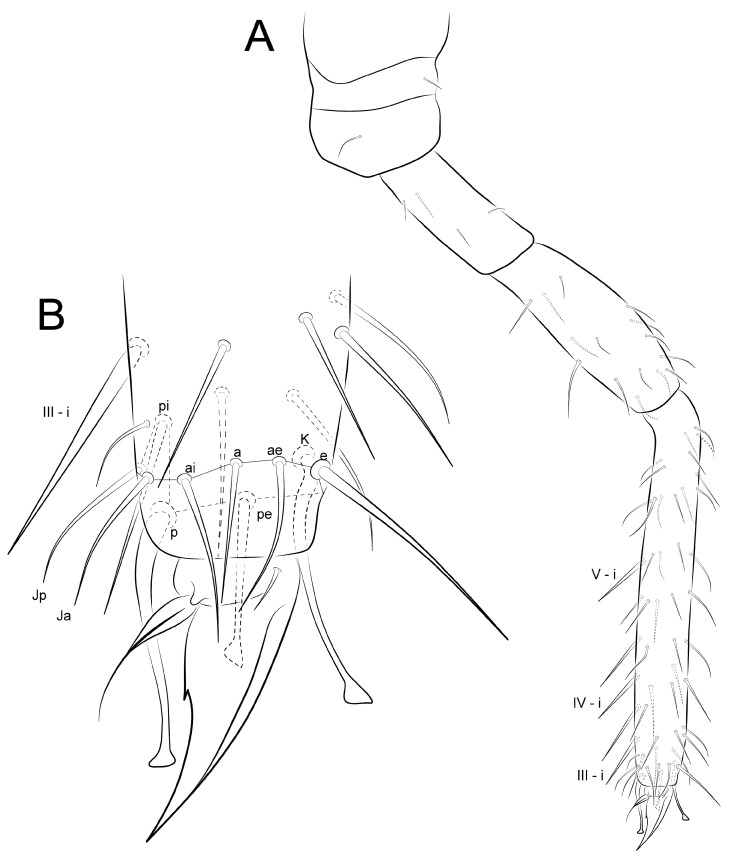
*Arlesminthurus caatinguensis* sp. nov. male’s leg I (anterior view): (**A**) complete chaetotaxy; (**B**) distal tibiotarsus and empodial complex.

**Figure 15:**
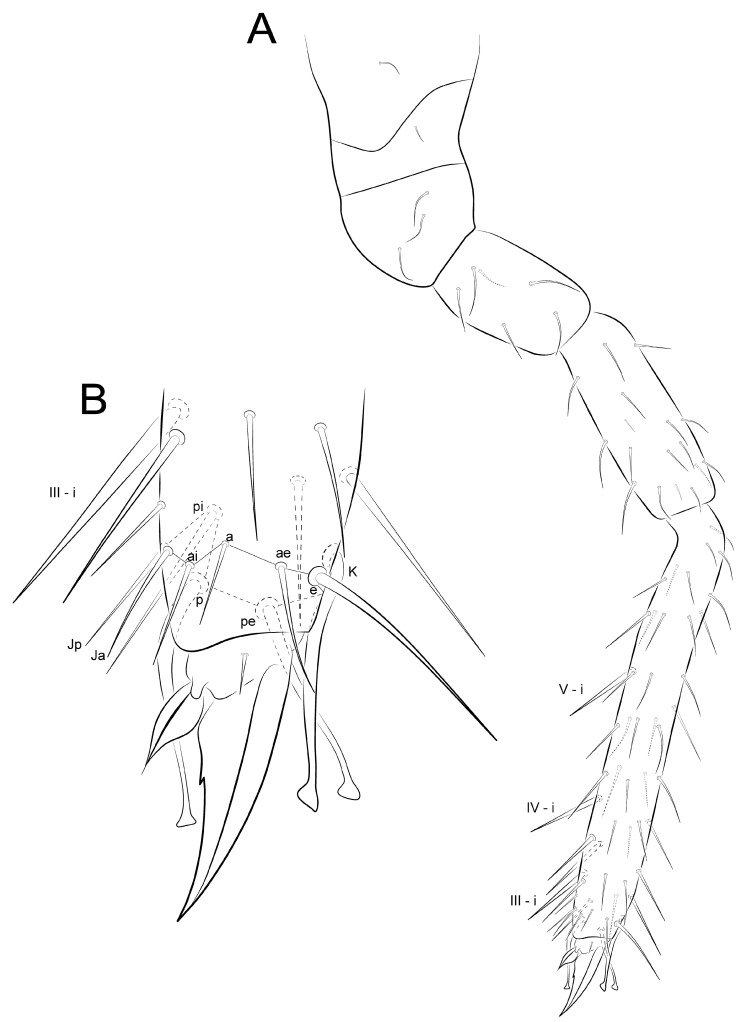
*Arlesminthurus caatinguensis* sp. nov. male’s leg II (anterior view): (**A**) complete chaetotaxy; (**B**) distal tibiotarsus and empodial complex.

**Figure 16:**
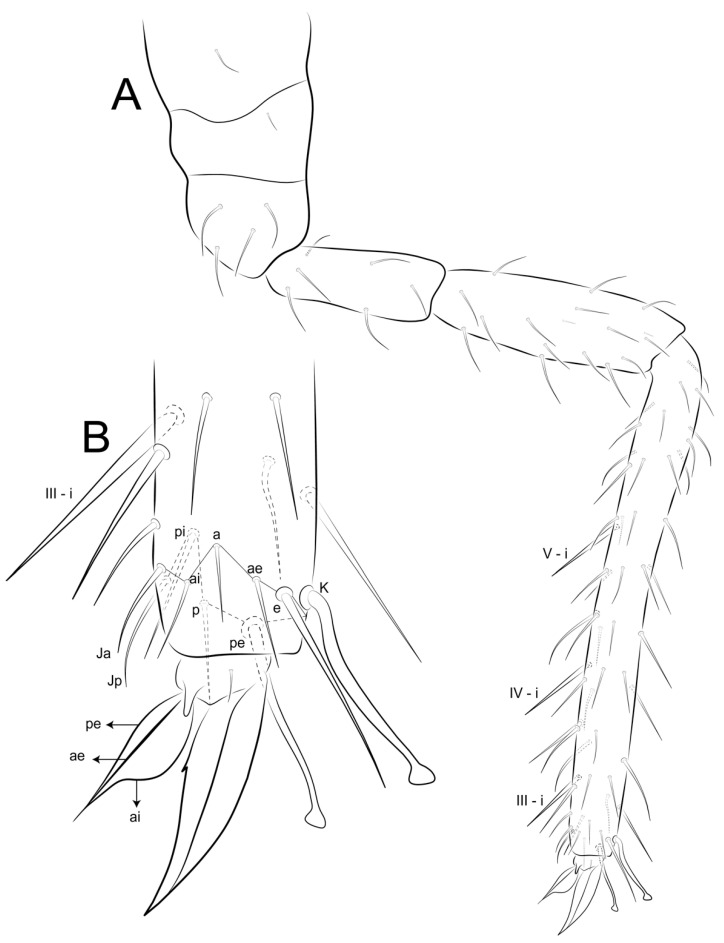
*Arlesminthurus caatinguensis* sp. nov. male’s leg III (anterior view): (**A**) complete chaetotaxy; (**B**) distal tibiotarsus and empodial complex.

**Figure 17:**
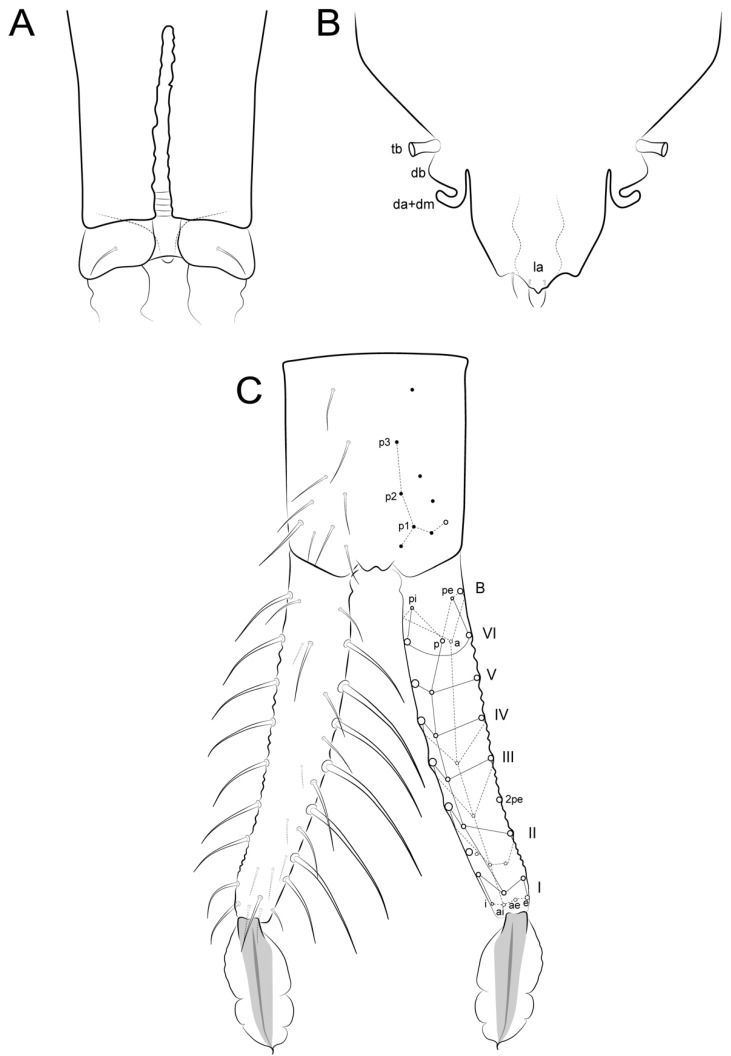
*Arlesminthuruscaatinguensis* sp. nov. male’s abdominal appendages: (**A**) collophore (anterior view); (**B**) tenaculum (anterior view); (**C**) furcula (dorsal view); the left side is presented as observed and on the right side is a schematic representation.

**Figure 18:**
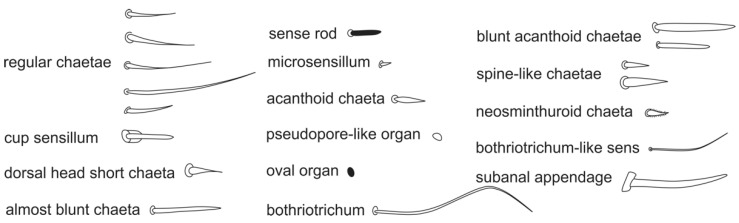
Chaetae symbols used in the chaetotaxy description of *Calvatomina gladiata* sp. nov.

**Figure 19:**
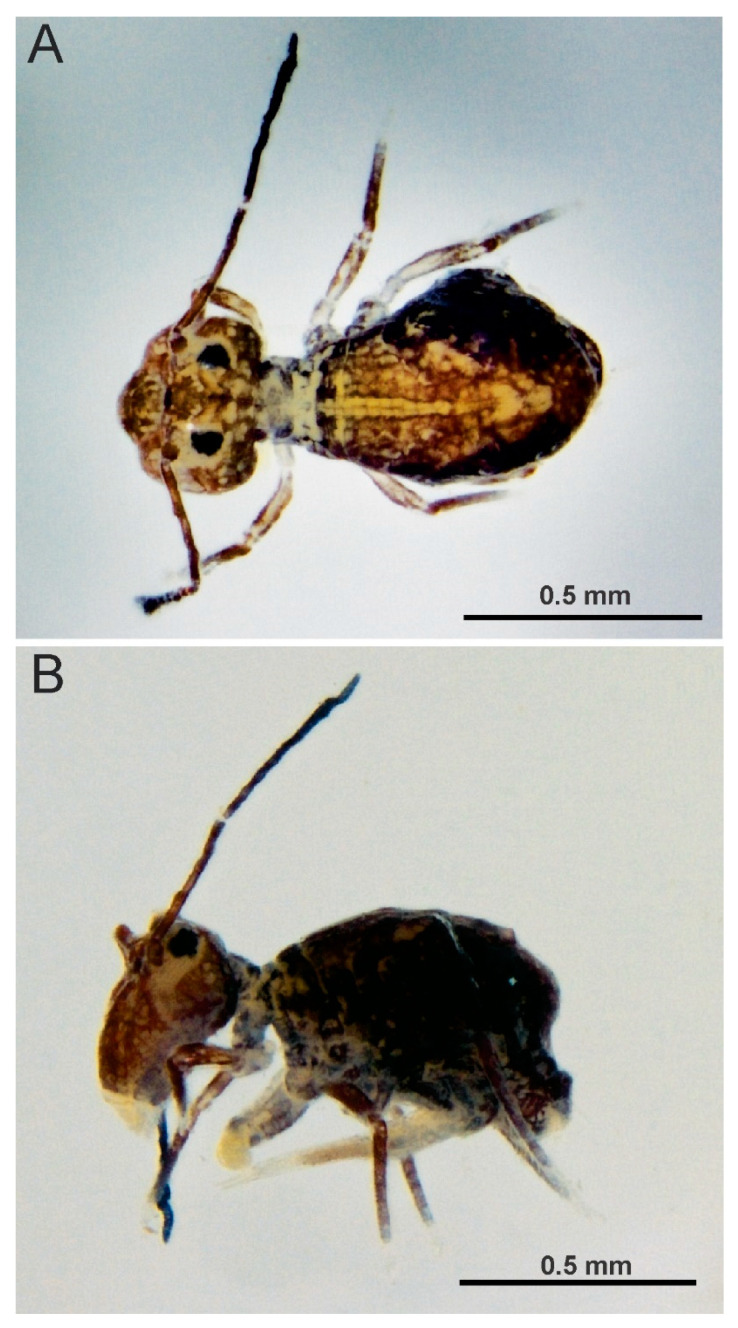
*Calvatomina gladiata* sp. nov. habitus in ethanol: (**A**) dorsal view; (**B**) lateral view.

**Figure 20:**
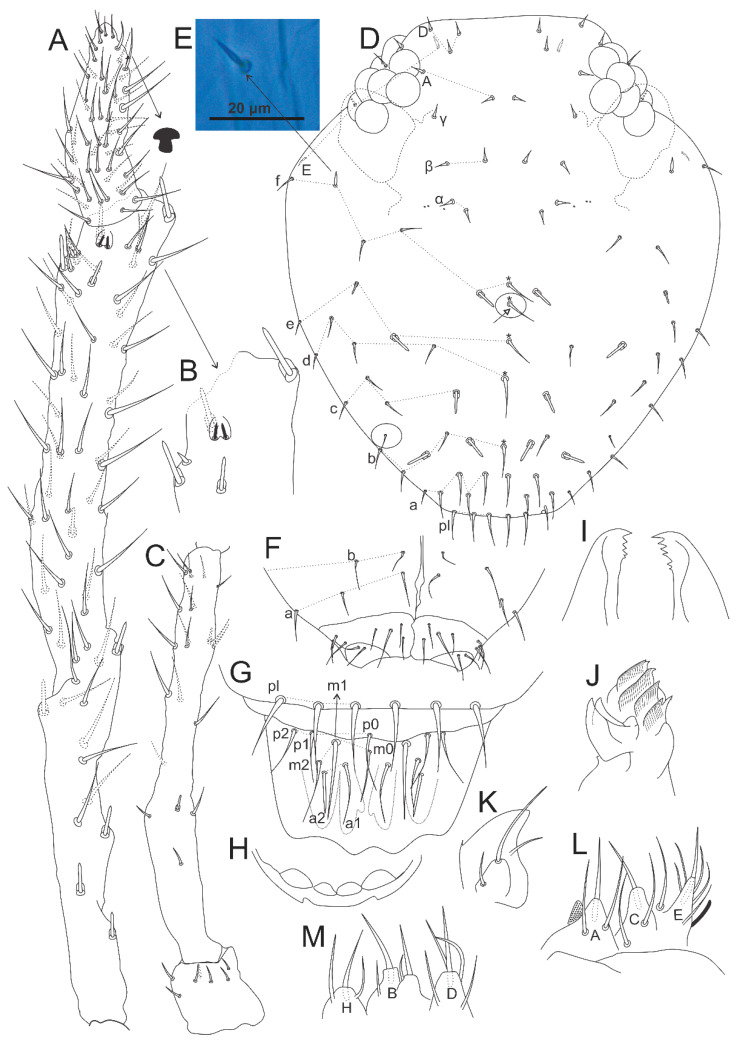
*Calvatomina gladiata* sp. nov. head: (**A**) Ant IV and Ant III ventral view (left side), arrow points to the subapical organ of Ant IV “mushroom-like”; (**B**) Ant III apex, with the apical organ, surrounding cup sensilla and microsensillum; (**C**) Ant II and I dorsal view (left side); (**D**) dorsal head chaetotaxy, antennal bases marked with dotted lines, circles indicate chaetae without clear homologies, asterisk indicate unpaired chaetae, white arrow indicates a chaeta present or absent; (**E**) acanthoid chaeta of **f** line; (**F**) ventral head chaetotaxy including basal labium (basomedian and basolateral fields); (**G**) prelabral and labral chaetotaxy; (**H**) labral papillae region; (**I**) mandibles apex (ventral view); (**J**) left maxilla capitulum (ventral view); (**K**) left maxillary outer lobe and sublobal plate; (**L**) labial palp papillae A, C and E and proximal chaetae (right side); (**M**) labial palp papillae H, B and D (right side).

**Figure 21:**
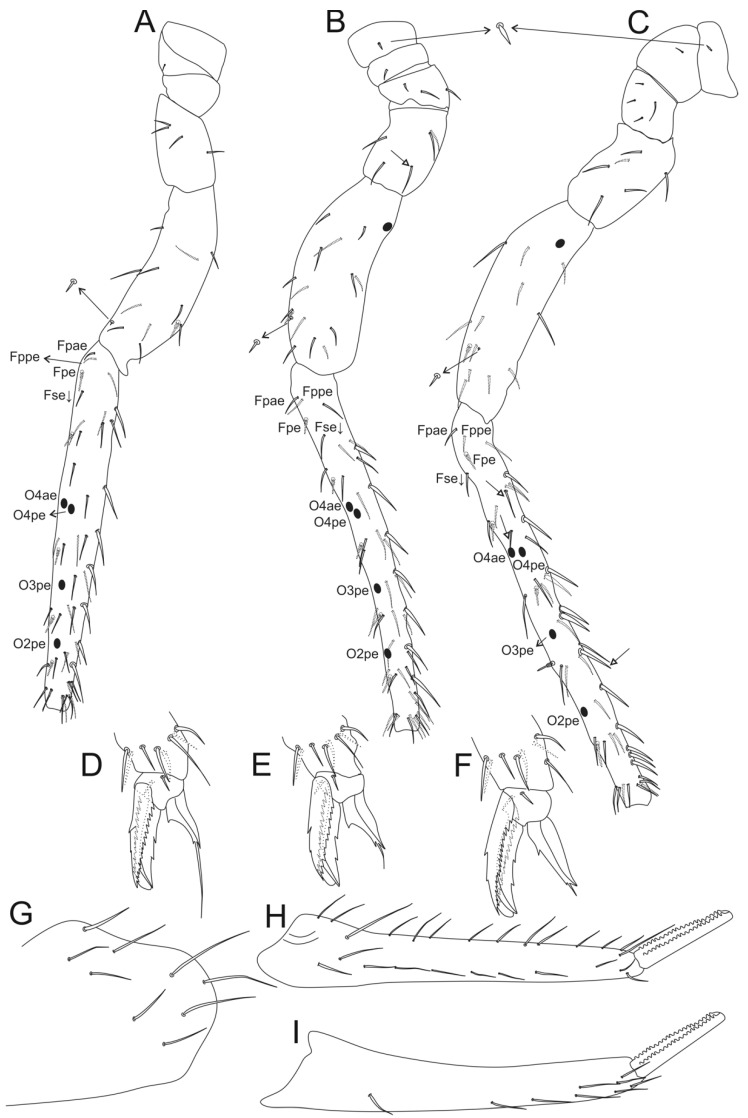
*Calvatomina gladiata* sp. nov. legs and furcula: (**A**–**C**) leg I–III, respectively (anterior view), black circles indicate oval organs, white arrows indicate chaetae present or absent, in detail micro and acanthoid chaetae; (**D**–**F**) empodial complexes I–III, respectively (anterior view); (**G**) manubrium (dorsal side); (**H**) dorsal dens chaetotaxy and mucro (inner view); (**I**) ventral dens chaetotaxy and mucro (outer view).

**Figure 22:**
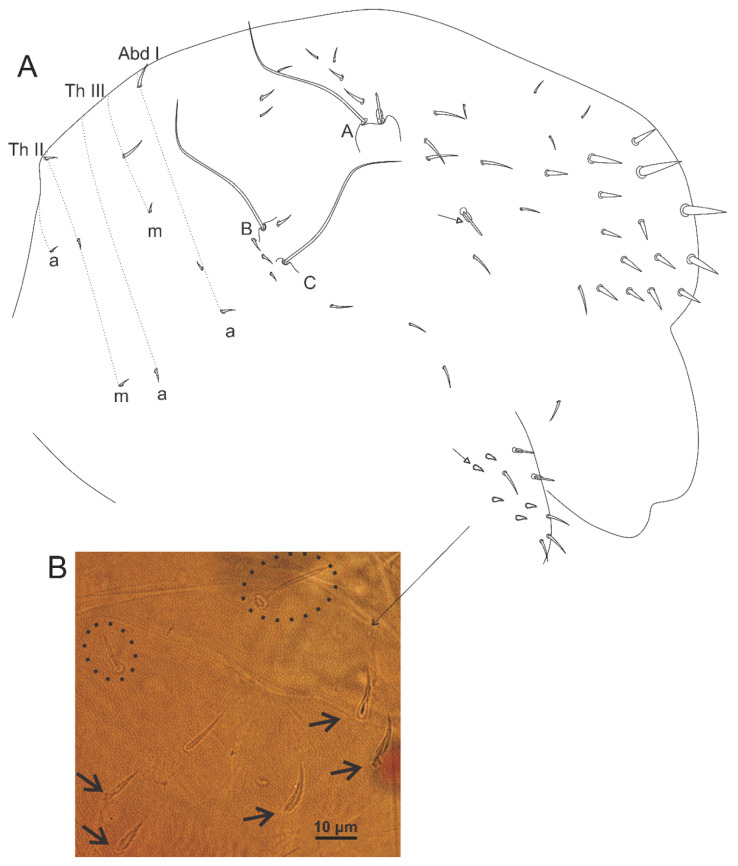
*Calvatomina gladiata* sp. nov. large abdomen (lateral view): (**A**) full chaetotaxy; white arrows indicate chaetae present or absent; (**B**) parafurcal area, circles indicate cup sensilla; arrows indicate neosminthuroid chaetae.

**Figure 23:**
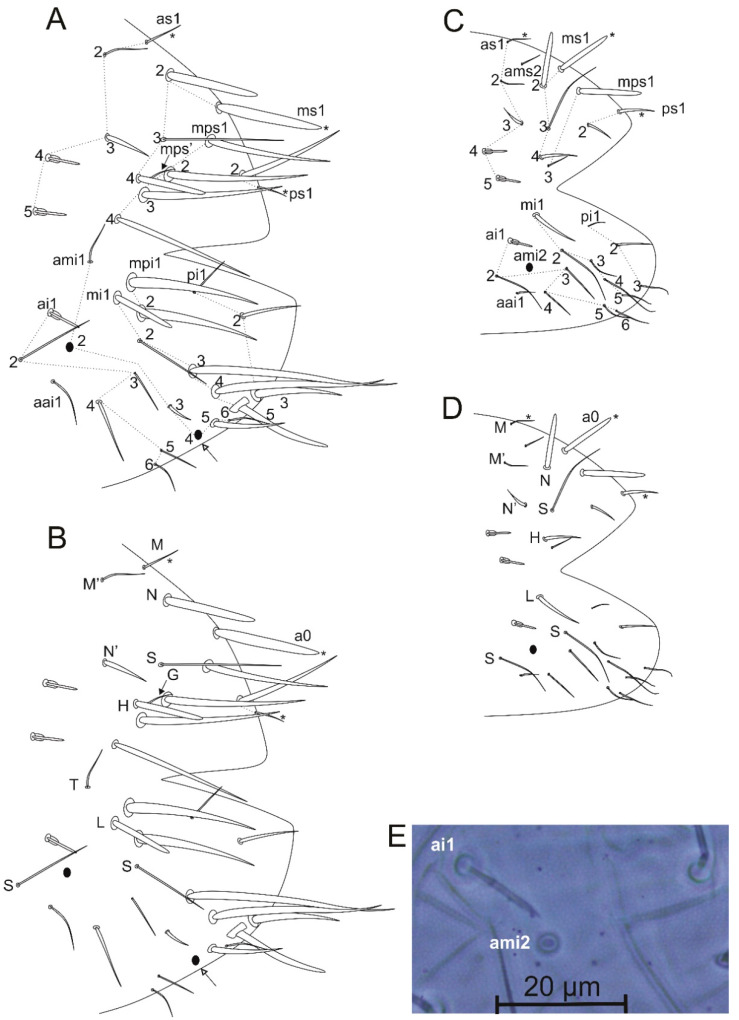
*Calvatomina gladiata* sp. nov. small abdomen (lateral view): (**A**) female (labels following Betsch [31]); white arrow indicates an element present or absent; (**B**) female (labels following Nayrolles and Betsch [18]); (**C**) male (labels following Betsch [31]); (**D**) male (labels following Nayrolles and Betsch [18]); (**E**) oval organ (**ami2**) and cup sensillum (**ai1**) of female.

In addition to the S-sens of the large abdomen present in at least these five genera (*Arlesminthurus, Bourletiella, Deuterosminthurus, Fasciosminthurus* and *Heterosminthurus*), plus unguiculi and distal tibiotarsal spine morphology, such taxa share a denser chaetotaxy, with extra chaetae on the dorsal head (e.g., Figure 8A and Figure 9A) and large abdomen, Ant IV with subapical sensillum and tibiotarsi I–III with three, three and two capitate tenent hairs, respectively [4,36,57,58].

Thus, it is fairly evident that these genera are similar to each other, and for that reason some species were transferred among them according to the evolution of Bourletiellidae’s internal taxonomy [4,11,12,36,57,58]. To go further in looking for similarities among *Arlesminthurus* species it is necessary to investigate if the bothriotrichum-like sens is also present in the other species of the genus, as well as the S-sens in other species of the Bourletiellidae genera, and to make sure whether both structures share the same morphology and could be considered as the same. We believe this feature could carry a strong phylogenetic signal within the Bourletiellidae.

### 4.2. *On the Differential Chaetotaxy of* Calvatomina *Species*

As noted by Yosii [8], the chaetotaxy of the small abdomen of females is the main feature used to separate *Calvatomina* species, as the color patterns could be insufficient or misleading. The displacement and morphology of such chaetae are of diagnostic importance among most Appendiciphora [3,4,36,43], and they were used in posterior studies not only to distinguish *Calvatomina* taxa, but also to divide the genus into species groups [4,18,37,60]. Another important diagnostic feature noted by Yosii [37] was the ventral chaetotaxy of the dens. Although the number of apical chaetae may be subject to different interpretations, the number and position of basal unpaired chaetae (1,1,0,0,1 or 1,1,1,0,1) provide clear data to compare the species, and they were used by Yosii [37] to separate the *bougainvilleae*-group and *Pseudodicyrtomina* Stach, 1957 [60] from the *rufescens* and *formosana* groups (a further discussion on such groups is presented in the next topic). The comparative Table 2 endorses such data as important diagnostic features.

Our revision of the Neotropical *Calvatomina*, specially the comparison between detailed described species as *C. gladiata* sp. nov. and *C. guyanensis*, suggested other easily accessible data which are useful in order to compare them. The number of cup sensilla on Ant. II, III and the clypeal area, the presence of a short acanthoid chaeta lateral to the antennal basis and even the number of clypeal unpaired chaetae proved to be helpful features to distinguish the species (see Table 2). Since the head chaetotaxy of the Dicyrtomidae is remarkably easier to understand compared to other taxa like Sminthurinae, due to the presence of fewer chaetae [30], the clypeal data can be used as reliable and clear diagnostic elements. Our previous studies also suggested that the morphology of the maxillary outer lobe and the sublobal plate and the number of proximal labial chaetae can be useful in separating closely related genera of Symphypleona and even species within the same genus, a result seen in other revisions as well [25,43,61,62]. For this reason we included this information in the diagnosis of *C. gladiata* sp. nov and such data should be investigated in further species of the genus and of the Dicyrtomidae.

Other features which may be useful in delimiting *Calvatomina* species are the number of cup sensilla on the tibiotarsi (a characteristic we dismissed in Table 2 since it was not entirely available for any other compared species) and the number of ventral tube, tenaculum and manubrial chaetae. For instance, on the tibiotarsus I of the new species and *C. tesselata* (Snider, 1990) [63] there are five cup sensilla, whereas in *C. guyanensis* apparently there are four, as well as in *C. sylvestratilis* (Snider, 1990) [63], *C. bellingeri* (Snider, 1990) [63], *C. madestris* (Snider, 1990) [63] and *C. microdentata* (Snider, 1990) [63], and only three in *C. longidigita* (Snider, 1990) [63]. Furthermore, as pointed in Snider [63], the parafurcal area chaetotaxy may be quite variable among different species of the genus.

As discussed in Nayrolles and Betsch [18], the chaetotaxy of the appendages and at least of the small abdomen has additions during the development from juveniles to the adults of *Calvatomina*. Therefore, the reduction in the number of some elements, such as on the collophore, tenaculum and furcula, may represent a type of pedomorphosis, a hypothesis which should be investigated in further species other than *C. guyanensis*.

### 4.3. *On the Validity of* Calvatomina *Internal Groups*

Yosii [37] divided *Calvatomina* into three groups of species (*bougainvilleae, rufescens* and *formosana*). The sole feature to separate the *bougainvilleae*-group from the others was the dens with 1,1,1,0,1 proximal ventral chaetae (vs. 1,1,0,0,1 in the other two groups). Yosii also suggested that such groups represent natural taxa (Yosii [37], p. 226). However, the ventral dental chaetotaxy formula of the *bougainvilleae*-group is not exclusive to it, since it is shared with other Dicyrtominae, at least with *Pseudodicyrtomina* [4,37,60]. Among the species of the *bougainvilleae*-group there is no clear and exclusive pattern in the females’ small abdomen chaetotaxy. For instance, its species may present **ms1**(**a0**) as a blunt acanthoid (as in *C. christianseni* and *C. discolor* sensu Mari-Mutt) or as a regular pointed chaeta (as in *C. bougainvilleae*, see Yosii [39], p. 37 and Yosii [37], pp. 228–229). Thus, the morphology of **a0**, which is the sole feature proposed to separate the *rufescens* from the *formosana*-group, is polymorphic within the *bougainvilleae*-group. Our revision of the Neotropical species shows that there is no clear diagnostic feature shared between *C. discolor* sensu Mari-Mutt and *C. christianseni,* which are exclusive to this group, other than this ventral extra chaeta on the dens (see Table 2). The finding of *C. rufescens* (from the *rufescens*-group) and *C. discolor* sensu Mari-Mutt from Colombia, which are remarkably similar in several diagnostic characteristics, with the exception of **ms4**(**H**) and **mi1**(**L**) chaetae on the female’s small abdomen and ventral dens chaetotaxy, also suggest that species from different groups could actually be closely related (see Mari-Mutt [16], p. 379). Therefore, the separation of the *bougainvilleae*-group from the others based on a single dental ventral chaeta, without the support of any other clearly exclusive characteristic, may be arbitrary. As a matter of fact, Yosii [37] did not explain clearly why such characteristics were used to split *Calvatomina* into species-groups, other than their usability.

Distribution does not clearly support Yosii’s groups either. The three groups were found in the Neotropical Region: *C. discolor* and *C. christianseni* from the *bougainvilleae*-group; *C. nymphascopula*, with a similar color pattern to the previous species but from the *formosana*-group; and as remarked before, *C. rufescens*, *C. guyanensis* and *C. gladiata* sp. nov. from the *rufescens*-group. The Hawaiian species, although most likely introduced, are represented by the three groups as well [63,64,65], which is also the case for the *Calvatomina* fauna from the Solomon Islands [37]. With such an overlapping distribution, there is no clear biogeographic evidence supporting a vicariant speciation of the internal groups of *Calvatomina*.

In this sense and considering the previous notes, we believe the species groups of *Calvatomina* described by Yosii [37] could be artificial and must be studied from a phylogenetic perspective, even if these groups can be handy for comparisons of species.

## 5. Conclusions

After our study, *Arlesminthurus* now has five and *Calvatomina* has 35 described species, respectively, with all *Arlesminthurus* and six *Calvatomina* taxa recorded in Neotropical regions. We expect that the detailed analyses of the morphology of both species, especially of *Arlesminthurus caatinguensis* sp. nov., may provide further foundations for the comparative studies of the Bourletiellidae and Dicyrtomidae. The observation of *Arlesminthurus caatinguensis* sp. nov. large abdomen chaetotaxy, with a bothriotrichum-like sens which is quite likely homologous to the S-sens seen in other bourletiellid genera, reinforces the importance of the study of the whole body chaetotaxy for the Symphypleona, a feature that is dismissed in several taxonomic studies.

## Figures and Tables

**Table 1:** Comparison among *Arlesminthurus* species.

		Species					
		*A. aueti* [11,12]	*A. caatinguensis*	*A. franzkafkai* [13]	*A. richardsi* [12]	*A. salinensis* [12]	
		(Arlé, 1961)	sp. nov.	Palacios-Vargas and Cabrera, 2015	(Arlé, 1971)	(Arlé, 1971)	
	Records:	PA, MT	PB and RN	Moropotente	PA	PA	
Characteristics		Brazil	Brazil	Nicaragua	Brazil	Brazil	
Sexual dimorphism in color pattern		–	–	+	+	–	
Color pattern of males’ head		1 median	1 median	1 postocular and	two lateral	diffuse, occipital head	
		longitudinal strip	longitudinal strip	1 lateral bands	longitudinal band	depigmented	
Body color pattern of males		2 lateral spot and	2 lateral spot and	W–shaped spot	two lateral	depigmented	
		1 longitudinal strip	1 longitudinal strip	strongly pigmented	longitudinal bands		
		depigmented	depigmented				
Color pattern of female’s head		as of the males	as of the males	2 postocular spots	1 interantennal spots	as of the males	
Body color pattern of females		as of the males	as of the males	W–shaped spot weakly	3 spots depigmented	as of the males	
Legs and furcula pigmentation		–	+	–	–	–	
Ant IV subsegments		6–7	8	9–11	8	8	
Male’s **γ** frontal chaetae (inferior)		short and robust	short and robust	fringed apex	swollen apex	hooked apex	
Male’s **γ** frontal chaetae (superior)		swollen apex	hooked apex	fringed apex	swollen apex	hooked apex	
Male’s interocular spines		3	0–4	–	0 *	0 *	
Male’s interocular spine-like chaetae		?	5–9	4	0 *	0 *	
Male’s unpaired chaetae (**f** lines)		larger	normal	normal	normal	normal	
Large abdomen ventral chaetae		?	6–7	3	?	?	
Abd VI robust spine-like chaeta (**?**)		?	+	–	–	–	
Unguis inner tooth		–	+	–	vestigial	vestigial	
Unguis III pseudonychia		?	–	+	–	–	
Male’s genital plate		?	3 + 4–5	3 + 7 *	?	?	
Female’s genital plate		?	1 + 4–5	0 + 4	?	?	
Manubrium dorsal chaetae		?	9	6	?	?	
Dens chaetae (per line)	inner	7 larger	7 larger	6 gently larger	6 gently larger	7 larger	
	outer	8	8	8–9	8	8	
	dorsal	8	8	8	8	8	
	ventral formula	1 *,3,1,1…0	3,3,1,1…1	3,3,1,1…1	3,3,1,1…1	3,3,1,1…1	
Mucro shape		wide	wide	thin	wide	wide	
Mucro edges	inner	irregular	two invaginations	smooth	irregular	smooth	
	outer	two invaginations	two invaginations	smooth	irregular	four invaginations	

Legends: (*) doubtful characteristic; (+) present; (–) absent; (?) unknown. Abbreviations of Brazilian States: PA = Pará, PB = Paraíba, MT = Mato Grosso, RN = Rio Grande do Norte.

**Table 2:** Comparison among *Calvatomina* species from Neotropical Region plus species of the *rufescens*-group with the small abdomen of the female with **as1**(**M**), **as2**(**M’**), **as3**(**N’**) and **ami1**(**T**) as small regular pointed chaetae.

		Species								
		*C. christianseni*	*C. cruciata*	C. discolor **	*C. gladiata*	*C. guyanensis*	*C. nymphascopula*	*C. pallida*	C. rufescens **	*C. trivandrana*
	References:	[15]	[8]	[16,42]	sp. nov.	[18]	[17]	[8]	[16,17,18,40,41]	[38]
	Type Locality:	Suriname	India	Sweden	Brazil	French Guiana	Puerto Rico	India	Finland	India
Characteristics	Species-Group:	*bougainvilleae*	*rufescens*	*bougainvilleae*	*rufescens*	*rufescens*	*formosana*	*rufescens*	*rufescens*	*rufescens*
Head color pattern		irregular spots	2 dorso-lateral spots	irregular spots	mostly pigmented	diffuse	irregular spots	1 median spot	diffuse	irregular spots
Large abdomen color pattern	dorsally	2 dark knife-shaped forms	almost stripped + cross-shaped form	2 dark knife-shaped forms	light sword-shape form	irregular spots	2 dark knife-shaped forms	diffuse	light median stripe	median spots
	laterally	J-shaped spot	all pigmented	J-shaped spot	all pigmented	narrow dark band	irregular spots	diffuse	large dark band	irregular spots
Ant III cup sensilla		?	?	?	10	9	10	?	10	8
Ant II cup sensilla		?	?	?	2	3	?	?	3	3
Clypeal **f** line acanthoid chaeta		–	?	+	+	+	+	?	+^5^	?
Clypeal cup sensilla		8	?	8	6	5	5	?	8^5^	?
Clypeal unpaired chaetae (superior)		5	6	5	4–5	5	4–5	?	5^5^	5
Collophore chaetae		?	?	1	2	2	?	?	1	1
Tenaculum chaetae		?	?	1	2	2	?	?	1	2
Manubrial chaetae		?	9	10	9	9	?	?	10	9
Dens dorsal chaetae *		23	24	24	25	24	21	23	21–24	24
Dens ventral formula		4,2,1,1,1,0,1	3,2,1,1,0,0,1	3,2,1,1,1,0,1	4,2,1,1,0,0,1	3,2,1,1,0,0,1	4,2,1,1,0,0,1	3,2,1,1,0,0,1	3,2,1,1,0,0,1	2,2,1,1,0,0,1
Abdominal neosminthuroid chaetae		5	?	5	4–5	5	4	?	5	?
Small abdomen of the female	**as1** (**M**)	BA	RC	RC	RC	BA	RC	RC	RC/BA	RC
	**as2** (**M’**)	?	RC	RC	RC	BA	RC	RC	RC	RC
	**as3** (**N’**)	?	RC	RC	RC	RC	RC	RC	RC ***	RC
	**ms1** (**a0**)	BA	BA	BA	BA	BA	RC	BA	BA	BA
	**ms2** (**N**)	BA	BA	BA	BA	BA	RC	BA	BA	BA
	**ms4** (**H**)	BA	BA	RC	BA	BA	RC	RC	BA	BA
	**ami1** (**T**)	–?	RC	RC	RC	RC	RC	RC	RC	RC
	**mi1** (**L**)	BA	BA	RC	BA	BA	RC	RC	BA	BA

Legends: (+) present; (–) absent; (?) unknown/doubtful; (BA) blunt acanthoid chaeta; (RC) regular pointed chaeta; (*) we considered here the sum of the dorsal, inner and outer lines of dental chaetae; (**) species recorded in Neotropical region (Colombia) by Mari-Mutt (1987) but described from Europe; (***) regular or thick pointed chaeta. Species-groups based on Yosii 1969.

## Data Availability

All data is contained within the article. All biological material is deposited at CC/UFRN and INPA as previously stated.
